# Exploring pathway interactions in insulin resistant mouse liver

**DOI:** 10.1186/1752-0509-5-127

**Published:** 2011-08-15

**Authors:** Thomas Kelder, Lars Eijssen, Robert Kleemann, Marjan van Erk, Teake Kooistra, Chris Evelo

**Affiliations:** 1 Department of Bioinformatics, Maastricht University, Maastricht, The Netherlands; 2 TNO, Research group Microbiology & Systems Biology, Zeist, The Netherlands; 3 TNO, Metabolic Health Research, Leiden, The Netherlands

## Abstract

**Background:**

Complex phenotypes such as insulin resistance involve different biological pathways that may interact and influence each other. Interpretation of related experimental data would be facilitated by identifying relevant pathway interactions in the context of the dataset.

**Results:**

We developed an analysis approach to study interactions between pathways by integrating gene and protein interaction networks, biological pathway information and high-throughput data. This approach was applied to a transcriptomics dataset to investigate pathway interactions in insulin resistant mouse liver in response to a glucose challenge. We identified regulated pathway interactions at different time points following the glucose challenge and also studied the underlying protein interactions to find possible mechanisms and key proteins involved in pathway cross-talk. A large number of pathway interactions were found for the comparison between the two diet groups at t = 0. The initial response to the glucose challenge (t = 0.6) was typed by an acute stress response and pathway interactions showed large overlap between the two diet groups, while the pathway interaction networks for the late response were more dissimilar.

**Conclusions:**

Studying pathway interactions provides a new perspective on the data that complements established pathway analysis methods such as enrichment analysis. This study provided new insights in how interactions between pathways may be affected by insulin resistance. In addition, the analysis approach described here can be generally applied to different types of high-throughput data and will therefore be useful for analysis of other complex datasets as well.

## Background

Biological pathways provide a powerful medium to explore and reduce the complexity of large datasets. Pathways organize genes, proteins, metabolites and their interactions into functional groups, often visualized as diagrams or networks. A commonly employed analysis technique using pathways is enrichment analysis, where pathways are represented as gene sets and where the aim is to find those sets that are enriched with entities of interest, such as differentially expressed genes [1]. More recent techniques also include connectivity within a pathway to measure its impact [2]. Such techniques allow a researcher to get an overview of biological processes that are likely to play a role in the studied phenomenon. The result of enrichment analysis is a sorted list of pathways, which is easier to interpret than a list of thousands of individual significantly expressed genes. However, each pathway in this list is presented as an isolated entity, while in reality these pathways can interact, for example through interacting or shared proteins and metabolites. To aid further exploration and interpretation of gene set enrichment results, it would be useful to get insight in possible relations or interactions between pathways and how these are affected in the context of the studied phenotype.

One way to get insight in possible relationships between pathways is to look at their overlap in gene, protein or metabolite content. Pathways with a high overlap might be related by shared paths. Tools such as ClueGO [3] and EnrichmentMap [4] allow the user to convert the list of enriched pathways into a network by calculating overlap between the sets. We used another approach with bi-partite graphs to create a network based on overlap in significantly regulated genes [5].

Another more functionally based approach is to find possible pathway cross-talk by looking at protein interactions between pathways. Cross-talk allows multiple pathways to exchange signals and influence each other. For example, the P53 pathway can control the Cell Cycle pathway by regulating the expression of p21 and can itself be activated by several pathways, for example the MAPK pathway. Metabolic pathways may share enzymatic reactions and may influence each other by influencing the availability of a substrate. These forms of pathway cross-talk are highly context dependent, for example, interactions between the P53 pathway and Cell Cycle depend on several external stress factors such as DNA damage or oxidative stress. Previous studies have already explored this idea [6,7] by building a pathway cross-talk network based on direct interactions between the proteins in the pathways. Both studies were based on the assumption that a pair of pathways is likely to interact when a higher number of protein-protein interactions are found between them than would be expected by chance. The work of Li et al. resulted in a scale-free pathway cross-talk network in which pathways in the same broad functional category indeed cluster together. Transcriptomics data was integrated into this network by finding cliques (a subset of pathways in which every two pathways are connected) which contained highly enriched pathways. Huang et al. performed a similar study, but also considered overlapping proteins between the pathways and integrated transcriptomics data at the protein level by counting only interactions between proteins encoded by differentially expressed genes. This resulted in context specific networks, reflecting interactions between pathways within the context of the dataset.

In this study, we investigate interactions between pathways by finding regulated paths between pathways for a given transcriptomics dataset. While the methods of Li et al. and Huang et al. only consider direct protein interactions between pathways, considering paths spanning multiple interactions may detect indirect interactions as well. Indirect interactions consist of paths including one or more proteins that are not annotated to a pathway, but do have known interaction or binding partners. In case the majority of the genes encoding these proteins and their interacting partners in two different pathways would be differentially expressed in a given condition, this indicates a potentially relevant path through which these pathways interact. An algorithm to detect pairs of pathways that contain such a path would allow researchers to directly explore and visualize possible paths along which pathways might interact in a given context. Furthermore, the inclusion of proteins that have not yet been annotated to a pathway in the analysis makes it possible to look beyond well studied processes, increasing the chance of generating novel hypotheses.

We designed a method to detect both direct and indirect interactions between pathways and visualize the resulting paths and applied this method to a transcriptomics dataset from the European Nutrigenomics Organization (NuGO) PPS2 study [8] to investigate the response to a glucose challenge in liver samples from obese and insulin resistant, as well as normal mice. Insulin resistance is a complex disease, not limiting to metabolism, but also associated to for example inflammation [9]. Interactions between different pathways might be especially relevant in the context of such complex phenotypes. Using this dataset, we hope to gain insight in the regulated biological processes during the response to the metabolic stressor glucose and the influence of a pretreatment with a high-fat diet on this response.

## Results

### A novel approach to explore pathway interactions

To find potentially regulated paths between different pathways, we designed a method based on non-redundant shortest paths in a weighted graph. The required input data are:

1. A set of pathways, each pathway consisting of a collection of gene, protein and/or metabolite entities.

2. An interaction network, providing interactions or functional associations between genes, proteins and/or metabolites, which can be compiled from different resources.

3. A weight value for each edge in the interaction network, indicating how much an edge will contribute to the length of a path. This can be based on experimental data, for example the expression of the genes that the edge connects.

**Figure 1:**
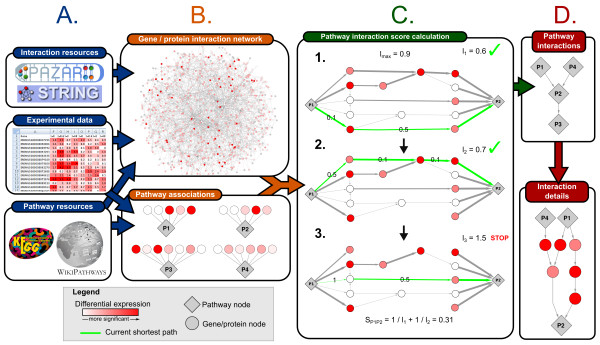
**Overview of the analysis approach to investigate interactions between pathways**. A: Information from different resources and experimental data is integrated into a weighted gene/protein interaction network and a set of pathways and their associated genes and proteins. B: Based on the interaction network, an interaction score and significance is calculated for each pathway pair. C: Example of the process of identifying a set of non-redundant shortest paths for the interaction of pathway P1 to P2. This panel shows step 5-7 of the calculation as described in the Methods section. D: Two representations of the resulting pathway interactions. The top panel shows the pathway interaction network, where each edge represents a significant interaction between two pathways. The bottom panel shows a detailed network showing the identified shortest paths between pathways P1, P4 and P2.

We will now briefly introduce the approach. For a more complete description of the procedure we refer to the methods section. For each pathway pair, a score is calculated by finding the set of non-redundant weighted shortest paths that have a length which is smaller than a given threshold and do not cross any other pathway. Non-redundant paths are used to identify the different routes of information transfer between the pathways. The length of each path is defined by the sum of the edge weights in the path. By assigning a lower weight for edges between nodes for which the corresponding genes are more differentially expressed in the dataset, paths that include regulated genes will get a shorter length. The score for a pathway pair depends on the number of paths found and their length (Figure 1C). The more and shorter the paths found between two pathways, the more likely it is that they interact and the higher the score. A pathway network is generated by creating an edge between a pathway pair if the p-value based on the score of that pair (see methods) is smaller than a given significance threshold.

This method was applied to pathways from the WikiPathways [10] and KEGG databases [11], which after merging strongly overlapping pathways resulted in a set of 236 pathways covering 6953 genes. A directed interaction network was generated based on reactions in these pathways and extended with reactions, functional associations between proteins, protein-protein interactions and transcription factor targets from several public databases (see methods). The resulting interaction network consisted of 6893 proteins and 138,105 interactions. Because a subset of interactions in this network have a specified direction (e.g. transcription factor targets), the identified interactions between pathways are directed as well.

### Application to an insulin resistance dataset

Pathway interactions were investigated in the context of a transcriptomics dataset of mouse liver samples. This dataset contains gene expression measurements before (t = 0) and at 0.6 hour (t = 0.6), 2 hours (t = 2) and 48 hours (t = 48) after a glucose challenge in two groups of mice fed either a low fat (LF) or high fat (HF) diet for 12 weeks before the challenge. After these 12 weeks all mice fed with HF diet had become obese and had developed insulin resistance [12]. To test for differential gene expression between different groups, in total 7 comparisons were made. Firstly, to determine the baseline effect of the different diets on gene expression, the measured genes were tested for differential expression between the HF and LF samples at t = 0. Secondly, to determine the changes in gene expression following the glucose challenge in each individual diet group, the genes were tested for differential expression between t = 0 and each other time point (t = 0.6, t = 2 and t = 48 hours). Each comparison resulted in a T-statistic for every gene representing the significance and direction of differential expression. The number of differentially expressed genes (q < 0.01) is shown in Table 1. For the number of differentially expressed genes in the glucose challenge response, the two diet groups display a different trend. In the LF group, the number of significant genes grows over time and is highest at t = 48, indicating that even 48 hours after the challenge gene regulation has not returned to the initial situation. However, in the HF group, the number of significant genes peaks at t = 2 and decreases again at t = 48. For each of the comparisons, pathways were identified that were enriched with differentially expressed genes (Additional file 1, Figure S1 and Table 1). The trend observed with respect to the number of differentially regulated genes in response to the glucose challenge is not apparent in the number of enriched pathways. Both the LF and HF groups show an increasing trend, where the number of enriched pathways at t = 48 is higher than preceding time points in both groups.

**Table 1:** Gene and pathway statistics for each comparison

Comparison	Significant genes, q < 0.01	Enriched pathways, p < 0.05	Edges with weight < = l_max_
**HF vs LF, t = 0**	1971 (17.3%)	54 (22.9%)	24055 (16.8%)

**LF t = 0 vs t = 0.6**	24 (0.2%)	17 (7.2%)	5054 (3.5%)

**LF t = 0 vs t = 2**	573 (5.0%)	43 (18.2%)	13251 (9.2%)

**LF t = 0 vs t = 48**	1607 (14.1%)	57 (24.15%)	20220 (14.1%)

**HF t = 0 vs t = 0.6**	773 (6.7%)	13 (5.5%)	15784 (11.0%)

**HF t = 0 vs t = 2**	2815 (24.7%)	17 (7.2%)	26845 (18.7%)

**HF t = 0 vs t = 48**	736 (6.4%)	40 (17.0%)	16857 (11.7%)

Number of significant genes, enriched pathways and edges in the interaction network that have a weight shorter than the maximal path length and hence contribute to the pathway interactions. Percentages are relative to the total number of measured genes, the total number of pathways and the total number of edges in the interaction network respectively.

Edge weights were derived from the computed gene-level T statistics of the comparisons to generate a weighted interaction network for each comparison (see methods). The number of edges in each interaction network that have a weight smaller than the maximum path length (see methods) and hence may contribute to a path between two pathways is shown in Table 1. These edges cover between 3.5% and almost 19% of the complete interaction network. The algorithm to detect pathway interactions was run for each comparison, resulting in 7 pathway interaction networks. A Cytoscape [13] session file that contains an interactive visualization for each network is available as Additional file 2.

The results of the algorithm can be represented as two different types of networks (Figure 1D). First are the pathway interaction networks that provide a global overview of how pathways might relate without showing the underlying protein interactions. Hence, each node in this pathway interaction network represents a pathway and each edge is a significant interaction between them. Second are detailed networks that also show the protein interactions that compose the paths between the pathways. These networks consist of two types of nodes, representing either a pathway or a protein. An edge between a pathway node and protein node represents association of the protein to the pathway and an edge between two protein nodes represents an interaction between these proteins. The next section provides an analysis of the generated pathway interaction networks by identifying pathways with a more central role in the network. This is followed by a global analysis of the protein interactions that form these pathway interactions, to identify proteins that may play an important role in pathway-crosstalk in this dataset. Finally, several potentially interesting pathway interactions will be highlighted and investigated more closely by zooming in to their protein interactions via the detailed network representation.

### Global analysis of the generated pathway interaction networks

The number of identified pathway interactions ranges from 25 to 207 across the different networks (Table 2). The differential expression between the HF and LF groups at t = 0 (before glucose challenge, but after 12 weeks of diet intervention) results in the most interactions, while the early response to the glucose challenge in the LF group results in the fewest interactions. For 66.7% of the pathways enriched with differentially expressed genes between the diet groups at t = 0, at least one significant interaction with another pathway could be found. This number is much lower for the early response to the glucose challenge in the LF group (20% and 27.9%) and HF group at t = 0.6 (23.1%).

**Table 2:** Statistics for each pathway interaction network

Comparison	Nodes (pathways > = 1 interaction)	Enriched nodes	Edges (p < 0.001)
**HF vs LF, t = 0**	123 (52.1%)	36 (66.7%)	207

**LF t = 0 vs t = 0.6**	17 (7.2%)	5 (20.0%)	25

**LF t = 0 vs t = 2**	57 (24.2%)	12 (27.9%)	52

**LF t = 0 vs t = 48**	82 (34.8%)	27 (47.4%)	108

**HF t = 0 vs t = 0.6**	43 (18.2%)	3 (23.1%)	54

**HF t = 0 vs t = 2**	111 (47.0%)	10 (58.8%)	160

**HF t = 0 vs t = 48**	82 (34.8%)	19 (47.5%)	92

The first column shows the comparison for which the network is generated. The second column shows the number of pathways in the network that have at least one significant interaction. The third column shows the number of pathways from the second column that are also enriched. The last column shows the number of significant pathway interactions. Percentages are relative to the total number of pathways and total number of enriched pathways respectively.

To identify potential focal pathways in the network, we can look at several node centrality measures. Additional file 1, Figure S2 shows the degree (number of interactions with other pathways) of the most connected pathways. Since the pathway interactions are directional, we can make the distinction between in-degree and out-degree. A pathway with a high in-degree is the target of many pathway interactions, which may indicate that the pathway is strongly regulated. A pathway with a high out-degree is the source of many pathway interactions, which may indicate a role in regulation of different targets. The three pathways with the highest in-degree for the network resulting from the comparison between diets at t = 0 are three stress response and apoptosis related pathways: Oxidative damage and apoptosis (14 interactions), FAS pathway and stress induction of HSP70 (14 interactions) and Apoptosis and its regulation by HSP70 (10 interactions). The first two pathways share 11 neighbors among their incoming interactions and 7 neighbors are shared among all three pathways. Interestingly, 5 of these 7 shared neighbors are in the top pathways with highest out-degree and 4 of those are also enriched with differentially expressed genes (p < 0.05). For most of the pathways with the highest in-degree, the out-degree is zero or very low and the corresponding node is acting like a sink, or end point. The same holds the other way around, where pathways with the highest out-degree tend to act as a source. To find pathways that may have a gatekeeper role in the network, we can look at the betweenness centrality, which measures how often a pathway occurs on shortest paths between the other pathway nodes in the network. The higher the betweenness, the more the pathway can control interactions between other pathways in the network. Additional file 1, Figure S3 shows the betweenness centrality of the pathways with highest betweenness. Notable are the many cytokine related pathways that all have a high betweenness in the network for the response at t = 2 in the HF diet group. None of these centrality measures seem to correlate with enrichment of the pathway and a pathway does not have to be enriched with differentially expressed genes to exhibit a central position in the pathway interaction network.

### Protein interactions contributing to regulated paths between pathways

Studying the protein interactions and functional associations that form differentially regulated paths between pathways may provide insights in the mechanisms of interacting pathways and reveal potential key regulators of pathway cross-talk that play a role in the dataset. The number of paths contributing to each pathway interaction network ranges from 52 for the smallest network (LF response at t = 0.6) to over 1500 for the largest (comparison between diets at t = 0). Most paths consist of direct interactions between proteins in the interacting pathways (Additional file 1, Figure S4). Only 17% of the paths over all networks are indirect and have one intermediate protein, while only 1 path has two intermediate proteins. The network for the response in the LF group at t = 48 consists of almost 50% of indirect paths, a considerably larger part than the other networks.

There is a lot of overlap among the paths between pathways, since the number of unique protein interactions that form these paths is much lower than the number of paths itself (Table 3). For example, for the comparison between the diet groups at t = 0, the 1562 paths between pathways are formed by only 440 unique protein interactions. The high overlap of protein interactions in the paths between pathways indicates the presence of proteins that are involved in multiple paths and might act as key regulators of pathway interactions. Additional file 1, Table S1 shows the proteins that are involved in most pathway interactions per network. Most of these proteins participate in interactions with highly connected pathways and a large part is involved in well-known signaling processes such as *Kras *and *Mapk1*, transcription factors such as *Jun *and cytokines such as *Il1a *and *Il1b*. There is some overlap of these proteins between the different networks, but there are no proteins that are in the top ten for each time point. One protein that plays a significant role in multiple networks is *Pik3r1*, which is known to play a role in insulin signaling by activation through IRS-1 [14] and participates in most of the paths for the networks for the response in the HF group at t = 2 and t = 48.

**Table 3:** Unique proteins and protein interactions between pathways for each network

Comparison	Unique protein interactions	Number of unique proteins	Unique proteins not in a pathway
**HF vs LF, t = 0**	440	198	25

**LF t = 0 vs t = 0.6**	8	6	0

**LF t = 0 vs t = 2**	47	43	1

**LF t = 0 vs t = 48**	275	142	14

**HF t = 0 vs t = 0.6**	53	47	0

**HF t = 0 vs t = 2**	400	200	18

**HF t = 0 vs t = 48**	118	86	2

Number of unique proteins and protein interactions that contribute to the paths between pathways for each network.

**Figure 2:**
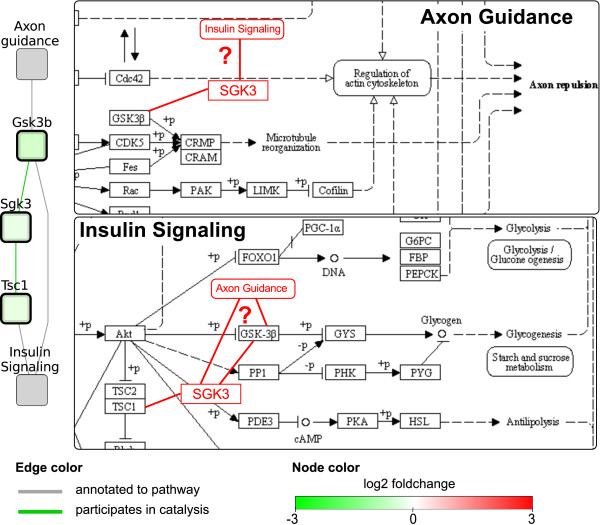
**An indirect interaction between the Axon Guidance and Insulin Signaling pathways in the network for the comparison between HF and LF diet at t = 0**. Left: Network representation of the identified path between the two pathways, consisting of three proteins *Gsk3b*, *Sgk3 *and *Tsc1*. Right: The location of these proteins in the KEGG pathway diagrams. The newly found indirect interactions have been added in red.

Proteins that act as intermediate in indirect interactions between pathways are of special interest. They are not annotated to any pathway yet, while they do seem to play a role in the context of this dataset, so studying their interactions may lead to new findings that would have been missed by looking at the annotations in the pathways alone. Table 3 lists the number of such proteins per network. Interestingly, while the network for LF at t = 48 contains relatively many indirect interactions, the number of proteins playing a role in indirect interactions is lower than expected. Upon closer inspection, the high number of indirect paths is mainly due to binding interactions of a single set of proteins (including differentially expressed *Mapre3*, *Cep57*, *Tubg1 *and *Nedd1*) that are not annotated to a pathway, but do all bind to a few proteins that play a role in many different pathway interactions, such as *Ywhab*, *Prkaca*, *Tubb5 *and *Hsp90ab1*. One of the proteins that is not annotated to any pathway is *Sgk3 *which plays a role in several pathway interactions for the response to the glucose challenge in the LF diet group at t = 2 and t = 48 (Additional file 1, Figure S5) as well as the comparison between the diet groups at t = 0 (Figure 2). In the latter, *Sgk3 *interacts with *Gsk3b *and *Tsc1 *to form one of the paths that form the identified interaction between the Axon guidance and Insulin signaling pathways. The genes encoding for these three proteins are all down-regulated in the HF diet group (q < 0.01). Since *Gsk3b *is present in both the Axon guidance and Insulin signaling pathways, in addition to the possible interaction between the two pathways via *Sgk3*, it might also form an alternative route within the Insulin signaling pathway that has been differentially regulated in the HF diet group (Figure 2).

### Visual exploration of the pathway interaction networks

The pathway interaction networks might be powerful tools for interactive exploratory analysis, by browsing and filtering the network visualizations in different ways to find interesting structures. The structural properties discussed above may guide to the most relevant parts of each network, which can then be explored in more detail. In this section we highlight several notable pathway interactions for each network and investigate the protein interactions that form the paths between the pathways.

### Differential expression between the LF and HF group

**Figure 3:**
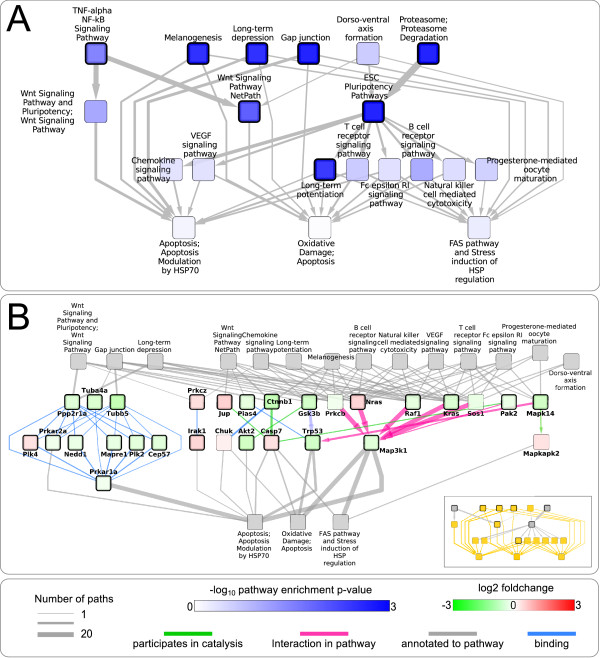
**Pathway interactions and detailed network visualization for the interactions with three apoptosis related pathways for the comparison between HF and LF diet at t = 0**. A: Subgraph of the pathway interaction network, based on incoming interactions to three stress response and apoptosis pathways with the highest in-degree. Pathway nodes with a thick border are significantly enriched (p < 0.05) with differentially expressed genes. B: The protein interactions that compose the interactions between the three apoptosis related pathways and their neighbors in the subgraph as shown in box A (see inset, included interactions are colored orange). Protein nodes have a thick border when their encoding genes are significantly differentially expressed (q < 0.05).

The network for the comparison between the diet groups at t = 0 may provide insight in the changes contributing to and resulting from the development of insulin resistance after feeding of a HF diet. Due to the large size of this network, and to aid visual exploration, we only include pathway interactions for which at least one of the participating pathways is significantly enriched (p < 0.05) with differentially expressed genes. Based on this filtered network, we study several potentially interesting interactions and subgraphs. A good starting point for this exploration might be the 3 apoptosis and stress response related pathways that have the highest in-degree in the network. Interestingly, 8 out of 14 pathways interacting with at least one of the three apoptosis related pathways also have an incoming interaction from the ESC pluripotency pathway, which is significantly enriched (p < 0.01). This pathway has a strong interaction (consisting of 38 paths) with the Proteasome pathway, which is also significantly enriched (p < 0.01). The remaining pathways interacting with the 3 apoptosis related pathways include 4 pathways with a relatively high out-degree of which 3 are significantly enriched (p < 0.05), and two versions of the Wnt signaling pathway, which both have a strong connection (25 and 27 paths) with the significantly enriched TNF-alpha NF-kB Signaling Pathway (p < 0.05). The subgraph containing these pathways and their interactions is shown in Figure 3A.

Based on this subgraph, we can "zoom in" to view the individual protein interactions that comprise the paths between the interacting pathways. For example, the paths between the 3 apoptosis related pathways and their neighbors are shown in Figure 3B. Despite the many neighbors of these three pathways, the number of unique proteins in these neighbors contributing to the interaction is relatively low (15 proteins for 14 pathways) and hence many of these proteins are annotated to more than one of the source pathways. This indicates the presence of common paths, shared by different pathway interactions. Indeed, there seem to be two main paths shared across different pathway interactions. Firstly, *Raf1*, *Kras*, *Nras *and *Prkcb *are present in more than 10 of the source pathways and all form a path to the three target pathways via *Map3k1*, which participates in 53 paths between the pathways. Secondly, there is a distinct path through *Prkar1a*, which forms an interface to the Gap junction, Long term depression and Wnt signaling pathway via indirect binding interactions with *Ppp2r1a*, *Tuba4a *and *Tubb5*. *Ppp2r1a *may also play a role in the interaction with the TNF-alpha NF-kB Signaling pathway and two versions of the Wnt pathway via indirect binding interactions with *Ywhab *(Additional file 1, Figure S6C). Since this is an indirect interaction, involving proteins that have not been annotated to one of the pathways, this is a target for further study to elucidate the potential role of these proteins in interaction between these two pathways. The link between the Proteasome and ESC pluripotency pathway can be largely explained by interactions between differentially expressed subunits of the proteasome and *Gsk3b*, *Apc *and *Ctnnb1 *(beta-catenin). The pathway diagram of the ESC pluripotency pathway (Additional file 1, Figure S7) reveals that these interactions are part of Wnt signaling and can influence the concentration of beta-catenin by targeting it for proteolysis after phosphorylation by *Gsk3b *and *Apc*. Interestingly, *Ctnnb1 *and *Gsk3b *are also present in other interactions, connecting the TNF-alpha NF-kB signaling pathway to the Wnt signaling pathways, the B-cell and T-cell receptor pathways to the Oxidative damage and apoptosis pathways via *Akt2*, *Casp7 *and *Trp53*, and the ESC pluripotency pathway to the B-cell and T-cell signaling and Chemokine signaling pathways via *Crk*, *Nfatc2 *and *Chuk*.

In addition to the pathways with a high degree, the network contains a potentially interesting component consisting of 10 metabolic pathways for which interactions with the Primary bile acid biosynthesis and Peroxisome pathways have been found. Out of the 12 pathways in this component, 9 are significantly enriched (p < 0.05). When looking at the protein interactions that form the interactions between these pathways, it turns out to be comprised of two binding interactions between *Hadh*, *Hsd17b10 *and *Hsd17b4 *(Additional file 1, Figure S7). These binding interactions are assigned by the STRING database [15] as subunits of a single enzyme complex, but in reality they are three distinct isozymes of 3-hydroxyacyl-CoA dehydrogenase [16,17]. Therefore, these proteins are probably not binding, but rather catalyzing a similar reaction in each of these pathways. While these proteins are differentially expressed and may play a role in insulin resistance and response to HF diet, they are not likely to contribute to an interaction between these pathways.

### Early response to the glucose challenge

The networks based on differential expression between t = 0 and each other time point after the glucose challenge in the LF group provide insight in pathway interactions that play a role during the response to the glucose bolus in mouse liver. The low differential expression at t = 0.6 in the LF group (only 24 significant genes, q < 0.05) is reflected in the small size of the corresponding network. Studying the protein interactions for that network reveals that all identified pathway interactions include *Jun *and *Fos*, for which the gene expression has both increased more than 5-fold compared to t = 0, and *Il1b *with a 2.5 fold increase (Additional file 1, Figure S8). Part of the interactions also includes *Il1a*, which is differentially expressed with a more than 2-fold increase. As a dimer, *Jun *and *Fos *form the AP-1 transcription factor, which responds to several stimuli including cytokines and controls several processes such as apoptosis and proliferation, for all of which related pathway interactions are identified. The network for the HF group at t = 0.6 shows a high overlap with the corresponding network for LF, including almost all edges of the LF network. The protein interactions and expression of Jun and Fos in the network for HF are similar to those in LF (Additional file 1, Figure S8), indicating that these interactions via AP-1 remain unaffected by obesity and insulin resistance. However, two additional interactions with the down regulated genes Irak4 and Il12a exist, suggesting a reduced capability to mount an inflammatory response since both factors are critically involved in innate immune responses. In addition to the largest component of the network for HF at t = 0.6, which is largely spanned by the interactions with *Jun *and *Fos*, there are several small components of isolated interactions between two or three pathways, which are not present in the LF network.

One of the components of the HF network at t = 0.6 that is not present in LF, for example, is composed of the Antigen processing and presentation and Spliceosome pathways, both interacting with the Lysosome pathway. These interactions have been identified because two genes encoding for proteins of the HSP70 family, *Hspa1a *and *Hspa1b*, are differentially expressed (> 4 fold) and interact with *Ap1s1*, *Ap1b1 *and *Cltc*. These proteins are involved in transport of lysosomal enzymes and show moderate, but not significantly differential expression (Additional file 1, Figure S9). Another potentially interesting component that consists of three pathways provides an interaction between the S1P receptor pathway, the Ribosomal proteins pathway and Insulin signaling pathway via *Mapk7*, *Nr4a1 *and *Sgk1*, which are all significantly up-regulated compared to t = 0 (Additional file 1, Figure S10), and *Rps6kb2*, which is up-regulated albeit not significantly (q = 0.066). Based on the pathway diagram, *Mapk7 *is a downstream target of the S1P receptor pathway, but because the other proteins in this pathway do not show significantly differential expression, it does not seem likely that the differential expression of *Mapk7 *is related to upstream activities in this pathway. However, since *Mapk7 *is present in the insulin signaling pathway as well, this might point to an alternative path within the insulin signaling cascade, which may be affected by HF diet in response to the glucose challenge.

The networks for t = 2 show much more difference between the HF and LF diet. There are only two overlapping pathway interactions and in addition, the network for LF is much less connected. A large part of the connectivity in the HF network situates around the merged version of the Cytokine-cytokine receptor interaction pathway and Cytokines and inflammation pathways (Cytokine pathway), which has the highest betweenness centrality. Several receptors listed in this pathway are differentially expressed, such as *Il1r1 *and *Il1r2*, involved in a path to Apoptosis and oxidative stress via *Casp8 *and *Casp9*. All other receptors that contribute to the pathway interactions, including up-regulated *Igf1r*, *Erbb3*, *Egfr *and down-regulated *Kdr *and *Fgfr4*, are receptor tyrosine kinases and form a path to several specific interleukin signaling pathways, T-cell and Kit receptor pathways via the *Cbl *protein. *Cbl *is a ubiquitin-protein ligase that can target these receptors for endocytosis [18]. Its expression profile shows a peak at t = 2 in the HF group only, where the expression has increased almost 2-fold compared to t = 0. We also found that the Endocytosis pathway, which indeed contains *Cbl *and the receptor tyrosine kinases as well, shares all its interacting pathways with the Cytokine pathway. In addition to *Cbl*, the interactions between the Endocytosis pathway and its neighbors also include the up-regulated *Cltc *and *Cltb*, which encode for the two light chains of clathrin, as well as several other proteins that are involved in clathrin dependent endocytosis (Additional file 1, Figure S11). This endocytosis mechanism is known to be responsible for transporting receptor tyrosine kinases to the endosome after ubiquination by *Cbl *[18]. Together, these interactions indicate that removal of these receptors from the cell membrane is affected at this point in the glucose challenge in the HF diet group specifically.

### Late response to the glucose challenge

A notable aspect of the late response to glucose challenge is that the LF group contains many more differentially expressed genes than at the early time points, while in the HF group the number peaks at t = 2 and is lowest at t = 48. This difference is not reflected in the pathway interaction networks for t = 48, which both contain almost the same number of nodes and edges. However, the overlap between these networks is small; only 4 edges are present in both networks, including the interaction between the TGF-beta receptor signaling Pathway and Proteasome. Interestingly, the proteins in the paths between these two pathways with the TGF-beta and Proteasome pathways behave differently between the HF and LF group (Additional file 1, Figure S12). All genes encoding the participating proteins in the Proteasome pathway are down-regulated in the LF network and up-regulated in the HF network. The proteins *Cdc27 *and *Ctnnb1 *in the TGF-beta pathway show a similar pattern. The interaction in the LF network contains another protein, *Axin1*, which is significantly up-regulated in the LF group and slightly down-regulated in the HF group is not included in the latter interaction due to its low significance.

The network for the LF group contains one tightly connected cluster, held together by the Antigen processing and presentation and T Cell receptor signaling pathways, including interactions with the enriched Cell cycle and TNF-alpha NF-kB signaling pathways. There are several central proteins in these interactions, including a set of shared binding partners between *Ywhab *in the Cell cycle and TNF-alpha pathways and *Tubb5 *in the T Cell Receptor pathway, or *Hsp90ab1 *in the Antigen processing pathway (Additional file 1, Figure S13). The *Gab1 *protein in the TNF-alpha pathway directly interacts with several proteins in the T Cell pathway, including the down-regulated *Fyn *and *Sos2*, and up-regulated *Pik3r2 *and *Pxn*. In addition, *Prkaca *is present in most neighbors of the Antigen processing and presentation and T Cell receptor signaling pathways, making it the protein involved in most paths for this network. Another interesting structure in the network is the second order neighborhood of the Insulin signaling pathway, containing the Wnt and Il-6 signaling pathways and the Starch and sucrose metabolism pathway, which are all significantly enriched (Additional file 1, Figure S14). A large part of the involved proteins are present in multiple of these pathways. For example, half of the proteins of the IL-6 pathway are also present in the Insulin signaling pathway. One of the proteins present in both the Wnt and IL-6 pathways, *Gsk3b*, forms an indirect path to the Insulin signaling pathway via *Sgk3 *which interacts with *Trib3 *and *Tsc1*, as well as a direct interaction via *Tsc1*. Two enzymes *Pygl *and *Gys2 *participate in the interactions with the Starch and sucrose metabolism pathway, interacting with several proteins in the Insulin, IL-6 and Wnt signaling pathways, which might indicate activated mechanisms that regulate these enzymes. The up-regulated *Pygl *gene encodes for glycogen phosphorylase which catalyzes the rate-limiting step in glycogen degradation. The down-regulated *Gys2 *gene encodes for glycogen synthase, involved in conversion from glucose to glycogen. Together, this indicates that at t = 48 the liver switches from storage of excess glucose to usage of the stored glycogen, possibly influenced by the pathway interactions identified here. The pathway interaction network for the HF response is typed by several key proteins including *Pik3r1*, *Kras*, and *Ctnnb1*. *Kras *and *Ctnnb1 *both have a protein interaction with *Pik3r1 *which plays a key role in many of the identified pathway interactions in this network. In addition, the up-regulated protein *Mapk14 *also interacts with *Kras *and 8 out of 12 pathways that contain *Mapk14 *also contain *Pik3r1*.

## Discussion

The goal of this study is two-fold. Firstly, we aimed to design methods for exploring possible pathway interactions in a specific context, which can be defined by an experimental dataset. Secondly, we explored pathway interactions relevant to glucose response in mouse liver and how these interactions are affected by high fat feeding-induced insulin resistance. We will first highlight several of the identified interactions and follow with a discussion about the described approach in general.

The high number of interactions with three apoptosis related pathways in the network for the comparison between diets at t = 0 suggest involvement of apoptosis in insulin resistant liver. Even though these pathways are not enriched with differentially expressed genes, they are the end point of many pathway interactions from upstream signaling pathways, which suggests that apoptosis is differentially regulated in the insulin resistant mice. Indeed, a relation between apoptosis and insulin resistance has been described before [19] and recent studies have shown that apoptosis might be increased in insulin resistant liver in humans [20,21]. The proteins identified could help to find mechanistic explanation for this observation, but it is hard to identify the most crucial step based on transcriptomics data alone, since many of the signaling events take place via post-transcriptional modifications.

The response to the glucose challenge at t = 0.6 in both LF and HF is characterized by the up-regulation of *Jun *and *Fos*, together forming the transcription factor AP-1, and *Il1a *and *Il1b*. This might indicate that AP-1 initiates several processes in the early response to glucose, possibly activated by *Il1*, and that this mechanism is not influenced by obesity or insulin resistance. A previous study has demonstrated that *Il1 *might indeed activate AP-1 in a hepatocyte cell line [22]. Immediate early genes such as *Jun *and *Fos *are genes that are activated transiently and rapidly in response to a wide variety of cellular stimuli, including glucose [23]. They represent a standing response mechanism that is activated at the transcription level in the first round of response to stimuli, before any new proteins are synthesized. Our interaction network contains 7 target genes of AP-1, of which 4 are present in the dataset, but none are differentially expressed during the early response. A possible cause might be that AP-1 is not activated and therefore its expression has no consequences, however we cannot determine this with the current dataset. Because of the general role of AP-1, there are probably more targets than annotated in our interaction network, so it may be of interest to analyze this effect in more detail using more specialized bioinformatics approaches or identify possible targets in a follow-up experiment. In addition to this shared mechanism, we also identified several interactions unique for the HF group.

First, the expression of two proteins of the HSP70 family show a distinct peak of over 4-fold increased expression at t = 0.6 in the HF group, but not in the LF group. We identified a possible interaction of these proteins with the Lysosome pathway that may indeed play a role in this pathway by uncoating of clathrin-coated vesicles [24]. However, based on our data it is hard to judge the relevance of this finding, since the three proteins in the Lysosome pathway that may interact with the HSP70 proteins show only moderate, not significantly differential expression at t = 0.6, their expression profiles do not correlate, and only a few additional genes in the Lysosome pathway itself show significantly differential expression at t = 0.6. It may be more likely that another function of these HSP70 proteins, for example their role as chaperones, could be related to their expression profile specific to the HF group. In addition, one of the two HSP70 proteins, *Hspa1a *(also known as HSP72), was found to play a role in preventing insulin resistance and blocking inflammation in human muscle by preventing c-jun amino terminal kinase (JNK) phosphorylation [25]. Since JNK can activate the c-Jun transcription factor, which was also found to be differentially expressed at t = 0.6, there may be a relation with the HSP70 proteins. Unfortunately neither the pathway collection nor the interaction network we used here contains any relevant interactions which are altered at the transcriptional level and could provide more insight in this possible relation.

Second, we identified an interaction between *Mapk7*, *Rps6kb *and *Sgk1 *in the Insulin signaling pathway and the transcription factor *Nr4a1 *in the Nuclear receptors pathway. Both *Nr4a1 *and *Sgk1 *show an expression profile with a peak at t = 0.6 in both the HF and LF group, however *Mapk7 *and *Rps6kb2 *are only up-regulated in the HF group. This difference might indicate the presence of an alternative mechanism through which *Nr4a1 *can be activated. *Nr4a1 *is a known transcriptional regulator of genes involved in glucose metabolism in mouse liver [26]. Because of the absence of any targets for this transcription factor in our interaction network we could not determine whether any downstream interactions to the Insulin signaling pathway are affected. Both the mRNA processing and Spliceosome pathways are significantly enriched for the differentially expressed genes at t = 0.6 and together with the presence of several differentially expressed transcription factors and the HSP70 chaperones this indicates that the response to the glucose challenge at t = 0.6 is mainly typed by a stress response and possibly transcriptional activation of genes to initiate the further response.

The most notable property of the pathway interaction networks for the glucose response at t = 2 is the high centrality of the Cytokine pathway. This pathway summarizes different cytokines and their receptors, and since several of these receptors are differentially expressed it makes sense that this pathway fulfills a central role in the network, by connecting to the different processes these receptors play a role in. We identified a regulated interaction between several cytokine receptor pathways and the Endocytosis pathway, which might point to increased removal of receptor tyrosine kinases from the cell membrane in the HF group after ubiquination with the up-regulated *Clb *protein. Several of the proteins participating in the interactions with the cytokine receptor and Endocytosis pathways are known to be related to insulin or even annotated to the Insulin signaling pathway. For example, *Cbl *plays an important role in the CAP/Cbl/TC10 dependent transport of GLUT4 vesicles [27]. This could point to an interaction with the Insulin signaling pathway, but we did not identify a significant interaction in our network. Upon closer inspection, however, an interaction with the Cytokine receptor pathway has been found, but is just above our significance threshold (p = 0.0018). This interaction involves *Cbl*, *Egfr *and several other proteins in the Insulin Signaling pathway, including three subunits of PI3K, *Crk *and *Igf1r*. Although this interaction was not considered significant, it might be biologically relevant given the high differential expression of *Cbl *and *Egfr*.

The switch from glycogen storage to glycogen breakdown observed at t = 48 can be expected after 48 hour following the glucose challenge. However, the link with the Insulin and Il-6 signaling pathways is interesting, since the Il-6 signaling pathway has already been linked to insulin actions and glycogen metabolism in vitro [28]. Another interesting observation is a difference in gene expression between the HF and LF groups in the interactions with the Proteasome pathway at t = 48, which is possibly related to the TGF-beta pathway. Degradation by the proteasome is an important mechanism in the TGF-beta pathway [29], which may be affected in different ways in the HF and LF groups. An identified interaction between the Wnt and Insulin signaling pathways might also be related to the observed changes in expression in the proteasome pathway, since *Gsk3b *is involved in degradation of beta-catenin by proteolysis, a process which can be inhibited by Wnt stimulation to stabilize beta-catenin, and which subsequently activates transcription of target genes [30]. Inspecting the expression profiles of *Ctnnb1 *and its associated proteasome related genes shows a different expression in the LF compared to the HF group at t = 48, so this mechanism could be affected by diet in the late response to the glucose challenge.

In summary, this analysis provided several new insights in the pathway interactions underlying insulin resistance in mouse liver and and the response to glucose. Initially the LF and HF group showed very distinct interactions with three apoptosis related pathways as most highly connected in the HF group. Despite the clear pathway and network differences in mouse livers after 12 weeks of LF or HF diet feeding, the initial response to a bolus of glucose is remarkably uniform and Il1a, Il1b as well as well as the transcription factors c-jun/fos (AP-1) appear to play an important role irrespectively of the degree of obesity and insulin resistance. This observation is in line with reports showing that the NLRP3 inflammasome senses glucose overload and triggers IL-1b activation in a caspase-3 dependent way [31] and suggests that this sensing mechanism is still active under HF diet conditions. An interesting observation is that there is much less overlap in the responses between LF and HF at later time points (> t = 2) and that more chronic types of inflammatory processes emerge. Of note, the peak of differentially expressed genes is at t = 2 in HF and at t = 48 in LF suggesting that, in the healthy state, the liver is able to cope with glucose overload by an extensive and permanent adaptation of its gene expression program while in HF livers the metabolic flexibility to switch the liver gene expression program seems to be reduced and genes associated with the TGF-beta pathway indicating liver stress and activation of fibrosis pathways.

As illustrated in the analysis described here, the method we developed is especially useful for exploring large and complex datasets to find interesting aspects and generate hypotheses. It can be used to add an extra dimension to differential gene expression and pathway enrichment results. Instead of redefining pathway boundaries based on an interaction network, this approach directly utilizes predefined pathways and thereby complements the search for activated or regulated genes and pathways by taking into account how they may interact. These interactions between pathways are not static and fixed, but are specific to the studied context, providing a dynamic view on the pathway landscape. This approach allowed us to explore the NuGO PPS dataset and identify relevant gene and protein interactions. It also helped to hypothesize about underlying mechanisms and possible downstream effects of certain groups of differentially regulated genes, thereby providing starting points for more focused follow-up studies or experiments.

During the analysis we mainly followed a fixed approach to explore the pathway interactions consisting of three main steps. First, we studied the pathway interaction network globally to identify highly connected or central pathways and proteins in the network. This, for example, pointed us to the apoptosis pathways in the comparison between the two diet groups at t = 0, which would not have been found by looking at pathway enrichment alone. In the second step, we interactively explored the interaction network using Cytoscape and zoomed in to specific pathway interactions or subgraphs by visualizing the protein interactions that form the paths between the pathways. This step is partly guided by the results of the previous step, since neighborhoods of the central pathways or proteins often point to interesting interactions. In addition, we looked for further interactions based on pathways that are known or expected to be involved in insulin resistance. For example, this lead us to the interactions with the insulin signaling pathways in the late HF response, which were not central in the network but appeared to be relevant because of enrichment of all involved pathways and the central role of insulin in this analysis. Finally, we often referred back to the original pathway diagrams to understand the context of the interacting proteins within the pathway and look for any upstream of downstream effects (Figure 2). Together, these steps helped us to better understand the generated pathway interaction networks and the biology behind the transcriptomics dataset.

In addition to the new perspective this analysis provides on the dataset, there are several advantages that make this method a useful complement to existing pathway analysis techniques. Firstly, compared to an analysis limited to pathway annotations, this combinatory analysis integrates additional information by using other sources of protein interactions and allowing indirect interactions including proteins that are not annotated to any pathway. This extends the coverage of the analysis with an additional 1660 genes that are in the interaction network but not annotated to any pathway, of which 929 are available in our transcriptomics dataset. This allowed us to move beyond well annotated knowledge in pathways, while still benefiting from the framework of canonical pathways and their intuitive visualizations. While the interaction network we used here is still relatively small compared to the number of interactions we expect to exist, current developments in measuring protein interactions [32] and identifying transcription factor targets [33] will further increase the coverage in the future. Secondly, when focusing on enrichment of gene sets or pathways alone, results might be missed since pathways may be relevant or activated without being enriched with significantly expressed genes, for example in case of post-translational regulation. For example, the three apoptosis related pathways identified in this analysis showed little differential expression, however given the number of other pathways it connects through via differentially expressed genes might indicate they may play a role in the context of this dataset. Finally, since genes and proteins often participate in different pathways and may have diverse functions, looking at individual pathway diagrams or lists of enriched pathways can be confusing or even misleading. Our method provides insight in the multiple roles of a protein in the context of the studied dataset. In the case where a gene is differentially expressed and also interacts with differentially expressed genes in multiple pathways, this will typically show up in the pathway interaction network, indicating that it acts on the verge of different pathways.

Besides several advantages, we also identified several possible improvements that may be made to this approach. False positive pathway interactions were identified on occasion. One cause can be falsely annotated interactions between proteins in the input interaction network. This is the main reason we already excluded associations based on text-mining only from the STRING database, however remaining data may still contain false associations. For example, the component of metabolic pathways positioned around the Primary bile acid biosynthesis pathway turned out to be a false result after close inspection, due to falsely associated enzymes in the STRING database. Over time, the quality of interaction resources will probably improve, also helped by curation initiatives such as WikiPathways.

Another cause of false or misleading results is the heterogeneity of many of the pathways. Pathway databases often include context specific pathways, which provide a summary of processes that are relevant to a specific disease or cell type or down-stream signaling of a specific messenger. However, the processes included in these pathways are often generic and play a role in other contexts as well, so finding an interaction with such a pathway does not automatically mean that its context is also relevant to the studied data. For example, we identified several interactions with the ESC Pluripotency pathway, which summarizes several signaling cascades regulating pluripotency in embryonic stem cells. It is very unlikely that embryonic stem cell functioning plays a role in our dataset, but still several interactions with this pathway were identified. These turned out to involve only specific parts of the signaling cascades, especially part of the Wnt signaling pathway. This is also a common problem in enrichment analysis, and our method does not provide a solution yet. Using a set of smallest generic core pathways with as little overlap as possible might lead to cleaner results. Possible alternative sources of pathway information that might provide better results than the databases used here could be Gene Ontology [34] or Reactome [35], which provide hierarchical gene sets and pathways, allowing us to move down in the hierarchy to more specific modules if necessary. However, this would require adaptations to the methodology in order to deal with this hierarchical structure. Altogether, these points show that the analysis approach presented here is mostly useful for exploratory analysis and offers only limited statistical proof for any of the findings. However, in the broader investigation of complex data towards more specific questions and testable hypothesis, this method provides a unique way to explore the data in a biological context.

The main shortcoming of using only transcriptomics datasets in pathway-based approaches is that these provide insight in changes at the mRNA level only. In this study, each identified pathway interaction is based on the assumption that if a group of interacting genes is consistently differentially expressed it is likely that changes are reflected at the protein level as well. However, this may not always be the case and it is not possible to investigate beyond this assumption using such datasets. In addition, a large part of signaling and pathway cross-talk probably works at a different level, through protein activation by phosphorylation or other post-translational (or post-transcriptional) regulatory mechanisms. If such interactions play a role, they cannot be identified with this dataset unless the interaction involves gene expression changes as well. The interactions that are most relevant to this dataset are transcriptional regulation, but unfortunately our interaction network contains relatively few transcription factor targets and we were unable to find a resource to increase this number in addition to the PAZAR database [36] used in this study. Despite this shortcoming, we were still able to show that it allows finding several relevant pathway interactions that help focus follow-up experiments.

Although in this particular study we used a transcriptomics dataset only, the method described here can directly be applied to other types of data or combinations of different data types as well. The method requires an edge weight for each interaction, which can be derived in different ways, depending on the type of interaction and available data. For example, the interaction network could be extended using the STITCH database [37], which defines interactions between chemicals and proteins. Thereby metabolomics data could be incorporated by providing weights for these interactions based on the metabolite abundance. In case both protein and metabolite abundance is measured, a weight could be calculated by combining the (differential) abundances of the two interaction participants, for example by taking the average. In addition, some types of data can even be used to define more specific edge weights, depending on what action the interaction represents. For example, if protein kinase activity measurements are available, this data could be used to obtain more accurate edge weights of specific protein interactions that represent activation. This could then even be combined with proteomics data to incorporate protein abundance in the edge weights as well and thereby obtaining more specific interactions by narrowing down the possible substrates of a protein kinase. Future publicly available datasets might cover these types of data at a larger scale and the method described here may prove useful in analyzing this complex data at a functional level. To this end, all scripts and input data used for this analysis are open-source and freely available (see Additional file 3).

## Conclusions

We designed an analysis approach to identify interactions between biological pathways in a specific context. By applying this method to a transcriptomics dataset, we identified relevant pathway interactions and possible key proteins involved in pathway cross-talk in the context of insulin resistant mouse liver, and at specific time points following a glucose challenge response. In addition, the analysis approach presented here can be applied to different types of high-throughput data and will be of more general use to facilitate interpretation of other complex datasets.

## Methods

### Microarray experiment design

The gene expression microarray data from the NuGO Proof of Principle Study 2 [8] was used. In this experiment, C57BL/6J mice were fed two different diets for 12 weeks, containing either 10% (LF) or 45% (HF) fat by energy. Animals fed the HF diet had developed insulin resistance by the end of this feeding trial. After 12 weeks, both diet groups were subjected to a glucose tolerance test and liver tissue was removed 0, 0.6, 2 and 48 hours after the glucose challenge and hybridized to NuGO Affymetrix Mouse GeneChip arrays (NuGO_Mm1a520177). The resulting dataset consists of 8 biological replicates per diet and time point (totalling 64 samples). The data was normalized using the GC-robust multi-array analysis (GCRMA) algorithm [38] and probesets were redefined and annotated to Entrez Gene identifiers using the Custom CDF version 11.0.2 [39]. Full details of the experiment and preprocessing of the microarrays are described in more detail elsewhere [8,12]. The resulting microarray dataset is available from the ArrayExpress repository (http://www.ebi.ac.uk/arrayexpress, accession number: E-MTAB-601).

### Microarray gene-level statistics

The different groups were statistically compared using the *limma *R package [40]. To study the initial difference between the two diets, before the glucose challenge, we tested for differential expression between HF and LF groups at t = 0. To study the response to the glucose challenge, we tested for differential expression between t = 0 and each other time point in the HF and LF groups. This resulted in the following statistics:

T_HF vs LF, t = 0_: The moderated t-statistic, representing the significance and direction of differential expression between the HF and LF group at t = 0.

T_LF, tx vs t0_: The moderated t-statistic, representing the significance and direction of differential expression between each time point after the challenge (t = 0.6, t = 2 or t = 48) and the time point just before the challenge (t = 0) in the LF group.

T_HF, tx vs t0_: The moderated t-statistic, representing the significance and direction of differential expression between each time point after the challenge (t = 0.6, t = 2 or t = 48) and the time point just before the challenge (t = 0) in the HF group.

Based on the corresponding p-values as provided by the *limma *package, q-values were calculated using the qvalue package [41] to correct for multiple testing.

### Pathways

Pathways from WikiPathways (analysis collection, v2010-12-07) [10] and KEGG (v2010-06-14) [11] were used. Pathways categorized under "Human diseases" at the KEGG website were excluded from the analysis.

Pathways with high overlap in protein content were merged into a single pathway using a step-wise procedure:

1. Calculate overlap between each pathway pair.

2. For each pathway, find other pathways that have > = 75% overlap and merge it with the most overlapping pathway.

3. Repeat step 1-2 until no pathway pair with > = 75% overlap is left.

This merging step was performed because pathways were combined from two different resources that partly overlap, so different pathways that actually represent the same underlying biological mechanism may occur.

### Pathway enrichment

To find pathways that are enriched with genes that score high in the differential expression test, the *geneSetTest *from the *limma *package was used. This test calculates a test statistic for each pathway by taking the mean of the absolute T-statistic of the genes in the pathway and calculates P-values by comparing the test statistic to an empirical null distribution based on 10,000 permutations with random gene sets. The resulting p-value for each pathway represents the significance of its enrichment with differentially expressed genes, regardless of their direction.

### Interaction network

The interaction network used in this study combines the following databases:

- Protein functional associations from the STRING database (v8.3) [15]. This database aggregates interactions from several protein-protein interaction repositories and pathway databases. Interactions based on text mining alone and those with a confidence score < 0.4 were excluded.

- Transcription factor targets from the PAZAR database (v2010-08-15) [36].

- Manually curated reactions and interactions from the pathways described in the section 'pathways'.

These interactions were merged into a single directed network. For interactions without a defined direction (e.g. binding), two directed edges in opposite direction were added.

### Gene and protein identifier mapping

Genes and proteins were mapped to a common database using the BridgeDb library [42] and accompanying synonym databases. All probesets in the transcriptomics dataset and genes and proteins in the interaction network were mapped to Ensembl gene identifiers using the Mm_Derby_20090720.bridge database. These mapped gene and protein identifiers will be referred to as *xref *in the following description of the algorithm.

### Finding interactions between pathways

To find possible interactions between pathways, we started by generating a unified graph, integrating the pathway and interaction information:

1. A graph *G_PX _*containing both the *xref *interactions and pathway-*xref *associations was created and reused for each group comparison. This graph contains two types of nodes, either representing an *xref *or a pathway. Edges were added between the *xref *nodes based on the interaction network. In addition, each pathway node was connected to each of the *xref *nodes that are associated with that pathway.

Then the following steps were followed for each group comparison separately:

2. Edge weights were calculated for *G_PX_*, based on the gene-level statistics described in the section 'Microarray gene-level statistics'. First, a transformation using a sigmoid function was applied to the T statistic:

This function ensures that the transformed values will range from 0 to 1 and results in a soft threshold (Additional file 1, Figure S16), where the μ determines the center of the threshold (the T value that will receive a weight of 0.5) and α determines the steepness of the curve (a higher α will result in a sharper cutoff). To transform the T statistic, μ = 3 and α = 2 were used. These parameters were chosen to emphasize genes with an absolute T statistic > = 3 (corresponding to unadjusted p-value < = 0.004) by receiving a lower weight.

Secondly, edge weights were assigned based on the transformed T statistic *f(T) *of the target *xref *nodes they connect to, or to the maximum value of 1 if no data was available for the target *xref*. If the target of an edge was a pathway node, the weight was set to 0. This way, for each edge, the weight value inversely depends on the value of the T statistic of the target gene, receiving a low weight value if the gene is differentially expressed. Thereby, paths that include genes (or the proteins they encode for) that are differentially expressed will have a shorter length.

**Figure 4:**
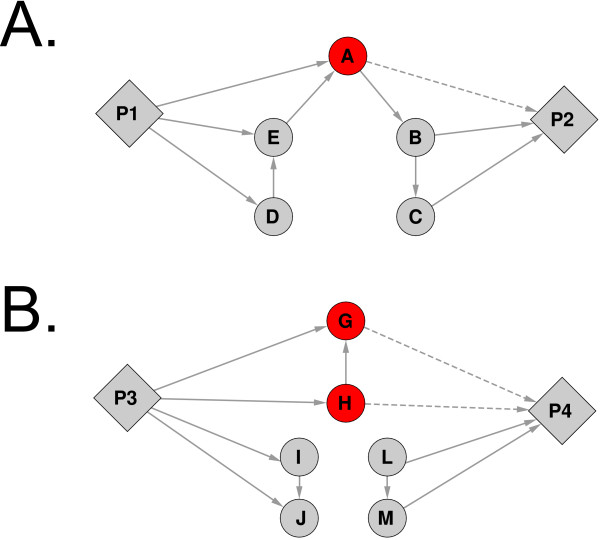
**Two examples of overlap between pathways with respect to the algorithm for finding pathway interactions**. Red nodes are proteins that are present in both pathways, dashed edges will be removed before finding shortest paths. A: A pathway pair with one overlapping protein (protein A) which connects to distinct paths within the two pathways. The overlap itself is not counted as interaction between the pathways, but by removing only the dashed edge A still acts as an indirect intermediate to establish a path between P1 and P2 via proteins B and E. B: A pathway pair which shares two proteins that do not connect to distinct paths within each pathway. In this case these proteins do not contribute to the pathway interaction, because the dashed edges are removed.

3. For each pathway node *Pn *in *G_PX _*a subgraph *G_Pn _*was created containing only nodes that could be reached within *nb *steps (visited edges) from *Pn*. In this study, parameter *nb *was set to 5, limiting the number of edges between *xref *nodes in a path to maximal three. This limit was introduced mainly to reduce calculation time. Based on the distribution of resulting path lengths, increasing *nb *is unlikely to have a large effect, as only a single path with three edges between *xref *nodes had a weighted length <*l_max_*.

4. For each pathway node *Pm *in *G_Pn _*another subgraph was created containing nodes *Pn*, *Pm*, the direct neighbors of *Pn *and *Pm *(the *xrefs *in these pathways) and all *xref *nodes that are not direct neighbors of a pathway node (*xrefs *that are not in any pathway). Excluding *xref *nodes that are present in pathways other than *Pm *and *Pn *is necessary to prevent detection of false interactions between *Pn *and *Pm *that cross other pathways. Such an interaction should not be represented as direct interaction between Pn and Pm, but rather as two interactions with the other pathway as intermediate between *Pn *and *Pm*. For *xref *nodes that were connected to both *Pn *and *Pm*, the edge to *Pm *was removed to prevent paths to go directly through an overlapping *xref *(Figure 4).

5. The shortest path between *Pn *and *Pm *in *G_Pn _*was found, taking into account the edge weights (the length of a path is the sum of its edge weights). The path length was stored and the edges of the path, except those that connect *Pn *or *Pm*, were deleted from *G_Pn _*(Figure 1C).

6. Step 5 was repeated until a preset maximum path length *l_max _*was reached (Figure 1C). The parameter l_max _controls how large the sum of the weights in a path may be in order to contribute to the score between two pathways. A larger l_max _will allow more interactions involving less significantly changed genes and result in more (but potentially less interesting) interactions, while a smaller l_max _will have the reverse effect and only include interactions with highly significantly changed genes. In this study, *l_max _*was set to 0.9, ensuring that paths between xrefs with low significance or no measured data (which will have a weight close to 1, see step 2) do not contribute to the interaction score.

7. For each pathway pair *P_n_P_m _*a list of path lengths *l_i _*is now available. These were converted to a single interaction score *S_PnPm _*for each pathway pair by calculating the sum of the inverted lengths:

8. Significance of the interaction was calculated by comparing the interaction score to an empirical null distribution. This null distribution was generated by repeating step 3-7 to recalculate an interaction score between *Pn *and *Pr *(a randomized version of *Pm*), resulting in a collection of randomized interaction scores *S_PnPr_*. For each permutation, *Pr *was generated by reconnecting the endpoints of its adjacent edges (which are the nodes representing the *xrefs *associated to the pathway) to different *xref *nodes from the interaction network that have a similar strength (also weighted degree [43]) as the original *xref *node. This way, the randomized pathway has a connectivity that is similar to the original pathway. Empirical p-values were calculated for each pathway pair by counting the number of times *S_PnPr _*is higher or equal to *S_PnPm _*and dividing this by the number of permutations. Firstly, p-values for each pathway pair were calculated using 100 permutations. Secondly, the resolution of the p-value of potentially significant pathway pairs was increased by adding another 1000 permutations for pairs that had a p-value < 0.1 after 100 permutations.

This algorithm was implemented in the statistical language *R *[44] and uses the *igraph *package [45] for handling graph structures and finding shortest paths between two nodes. The R source code and utility scripts to generate and format the data are available as Additional file 3.

To reduce the contribution of overlapping proteins between pathways to the interactions between pathways, the edge from an overlapping protein to one of the pathways in a pair is removed in step 4. While pathways with more than 75% overlap were merged, some pathways might still have much overlap that may outweigh the influence of pathway interactions via protein interactions. However, to completely discard overlapping genes might be too conservative, since an overlapping protein may be a key regulator that might connect two distinct parts of the different pathways. For example, in Figure 4A, two pathways both contain protein A, which connects to a distinct path in each pathway. If protein A would be excluded from the analysis (as in Li et al. [6]), a relevant interaction between the two distinct paths will be missed. However, in Figure 4B, two pathways share a complete path of three proteins which does not connect to any other distinct part within each pathway and hence these proteins are unlikely to contribute to an interaction between the pathways. In this case, our approach of removing one of the edges with the pathway will give equal results as excluding the proteins.

### Network analysis and visualization

All networks were visualized using Cytoscape [13]. Network properties such as average path length, clustering coefficient, node degree and betweenness centrality were calculated using the *igraph *library in R. Betweenness centrality as displayed in Additional file 1, Figure S3 was normalized by dividing the value calculated with the *igraph *function *betweenness *by the maximum centrality, which is the total number of node pairs in the network, excluding the node for which the betweenness centrality is calculated.

## List of abbreviations

HF: high fat (experimental group); LF: low fat (experimental group); NuGO: European Nutrigenomics Organization; PPS2: Proof of Principle Study 2.

## Authors' contributions

Designed and performed computational analysis: TK. Designed and performed transcriptomics experiment: MvE RK TeK. Normalization and quality control of transcriptomics data: MvE. Extensive reviewing and editing of the manuscript: CE, LE. Drafted the manuscript: TK. All authors read and approved the final manuscript.

## Supplementary Material

Additional file 1 **Supplementary figures and tables**. Contains the supplementary figures and tables. Click here for file

Additional file 2 **Cytoscape visualization of pathway interaction networks**. Contains the cytoscape session files with interactive visualizations of the pathway interaction networks. These can be viewed with the freely available Cytoscape software http://www.cytoscape.org/. Click here for file

Additional file 3 **Scripts and input data**. Contains instructions on how to obtain the scripts, utilities and formatted input data that was used in this analysis. Click here for file
