# Supplementary material for: Exploring pathway interactions in insulin resistant mouse liver
Source: BMC Syst Biol. 2011 Aug 15;5:127. doi: 10.1186/1752-0509-5-127 (PMC3169508; doi:10.1186/1752-0509-5-127)
Supplement: Additional file 1 — Supplementary figures and tables. Contains the supplementary figures and tables. [file 1752-0509-5-127-S1.PDF]

Supplementary figures

Pathway enrichment for each gene-level test

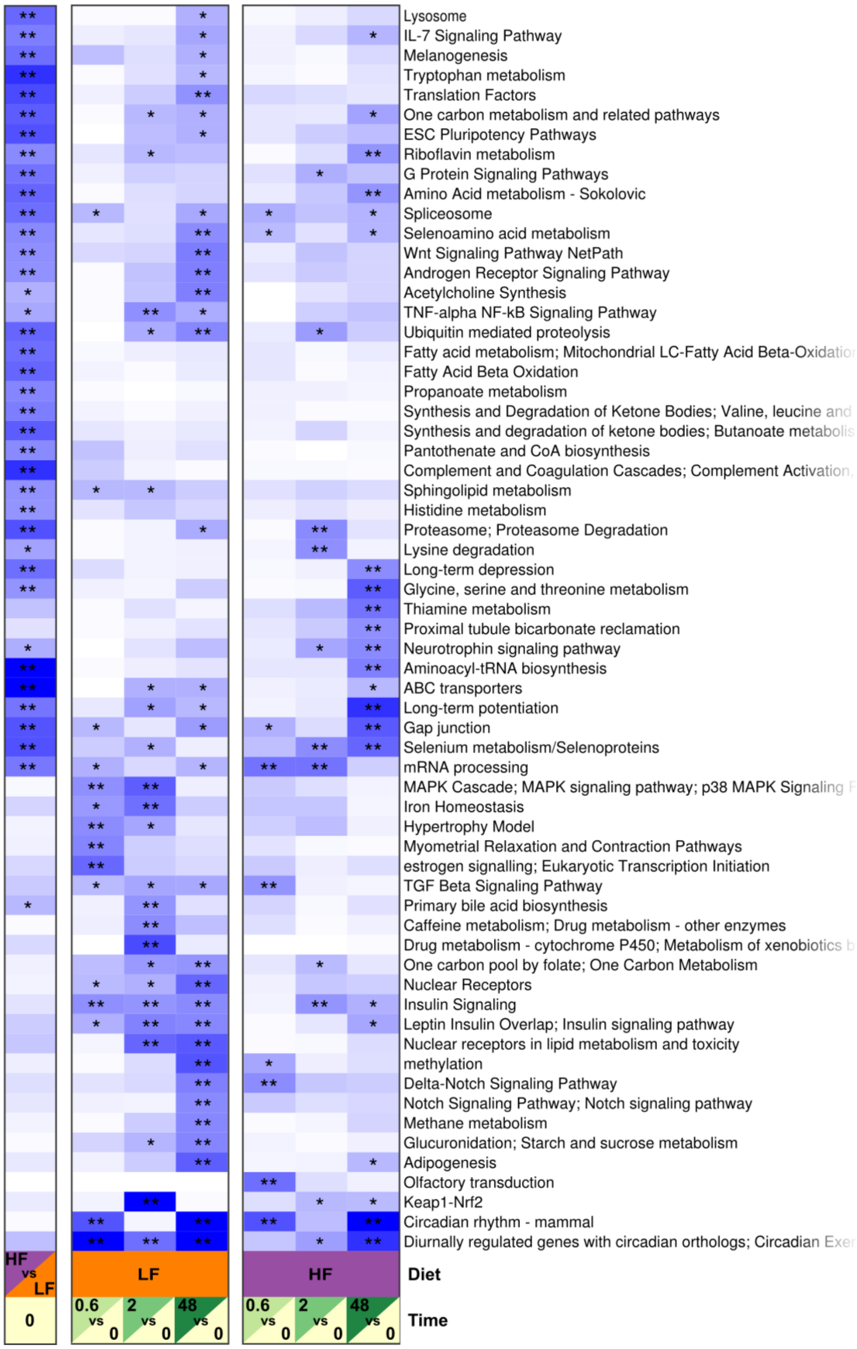

Supplementary figure 1: Pathway enrichment for each comparison. The first column shows enrichment with differentially expressed genes between the diet groups at t = 0, the second and thirds columns show enrichment with differentially expressed genes for t = 0 compared to the other time points in LF diet and HF diet respectively. Only pathways with an enrichment p-value < 0.01 in at least one of the tests are shown. The pathways are sorted by hierarchical clustering over all columns to group pathways with a similar response. Saturation of each box indicates enrichment significance, the more saturated blue, the more significant. In addition, the boxes are starred by significance threshold (* for p < 0.05, ** for p < 0.01).

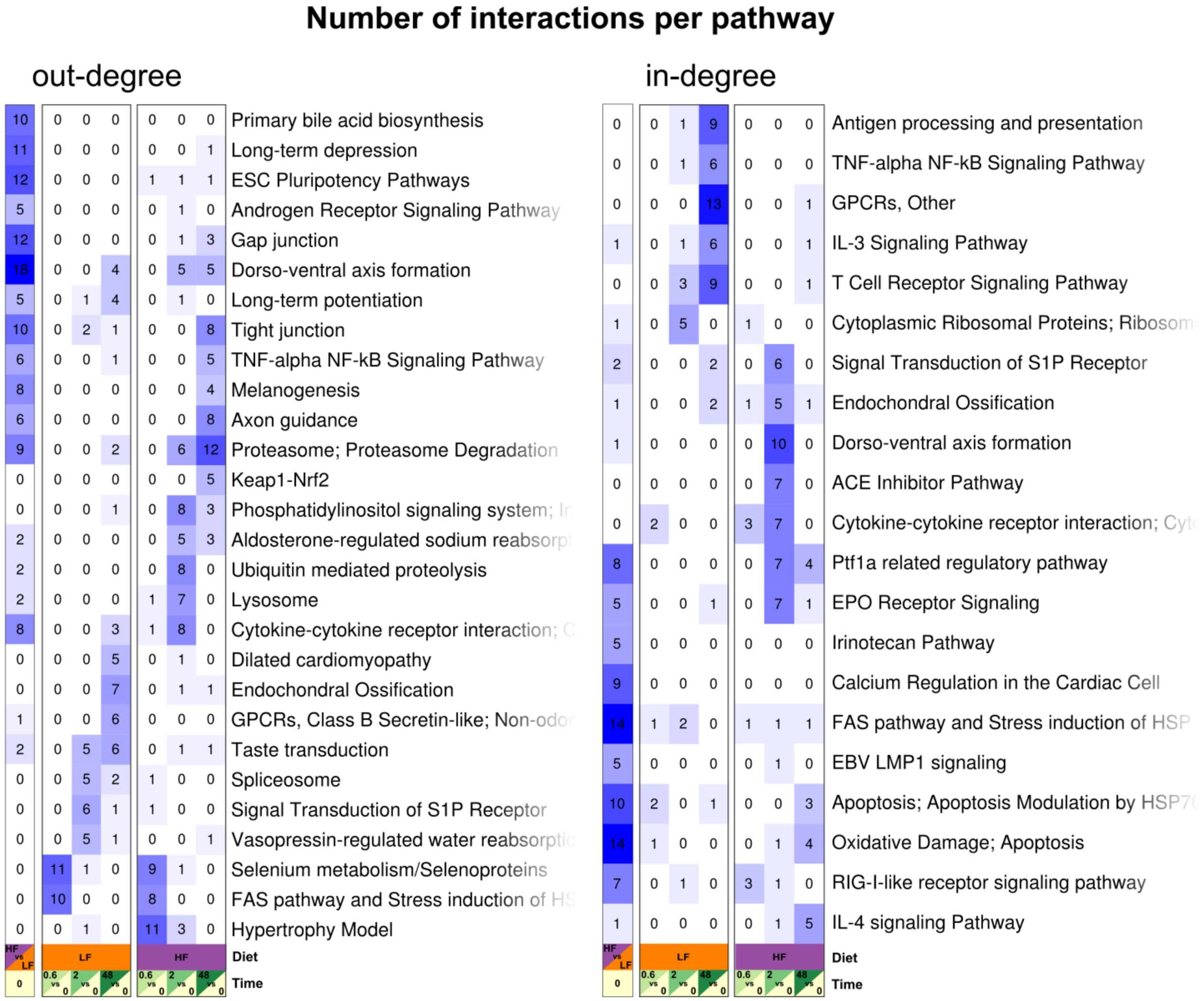

Supplementary figure 2: In and out degree (number of interactions) for the most connected pathways per network. Only pathways with a degree >=4 in at least one of the networks are included.

Normalized betweenness per pathway

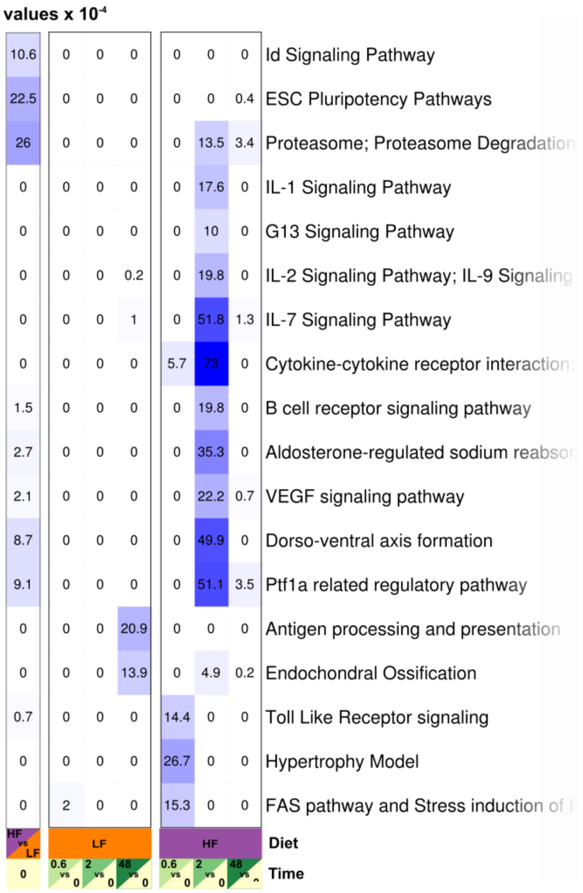

Supplementary figure 3: Normalized node betweenness centrality per pathway, which measures how often a node occurs on shortest paths between the other nodes in the network. The value drawn in each cell is the normalized by dividing by the maximum betweenness centrality (values multiplied by 10^4).

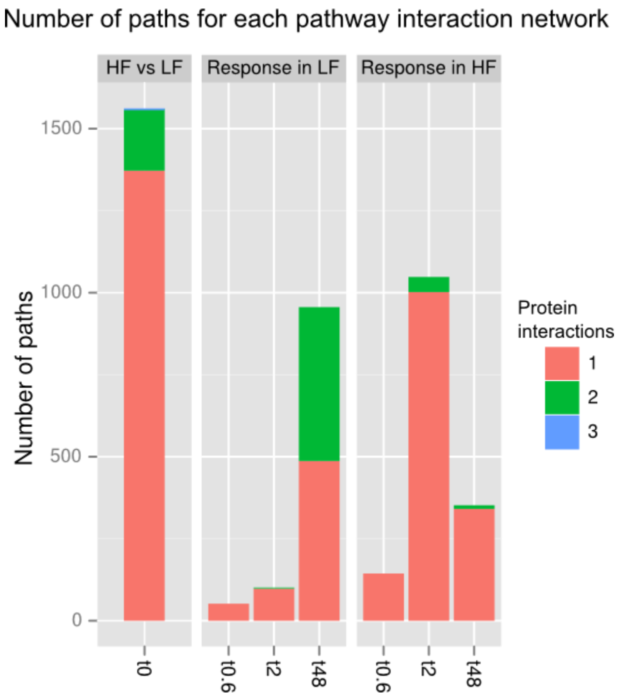

Supplementary figure 4: Total number of protein interaction paths between pathways for each pathway interaction network. Bars are colored by the length (number of interactions) of each path.

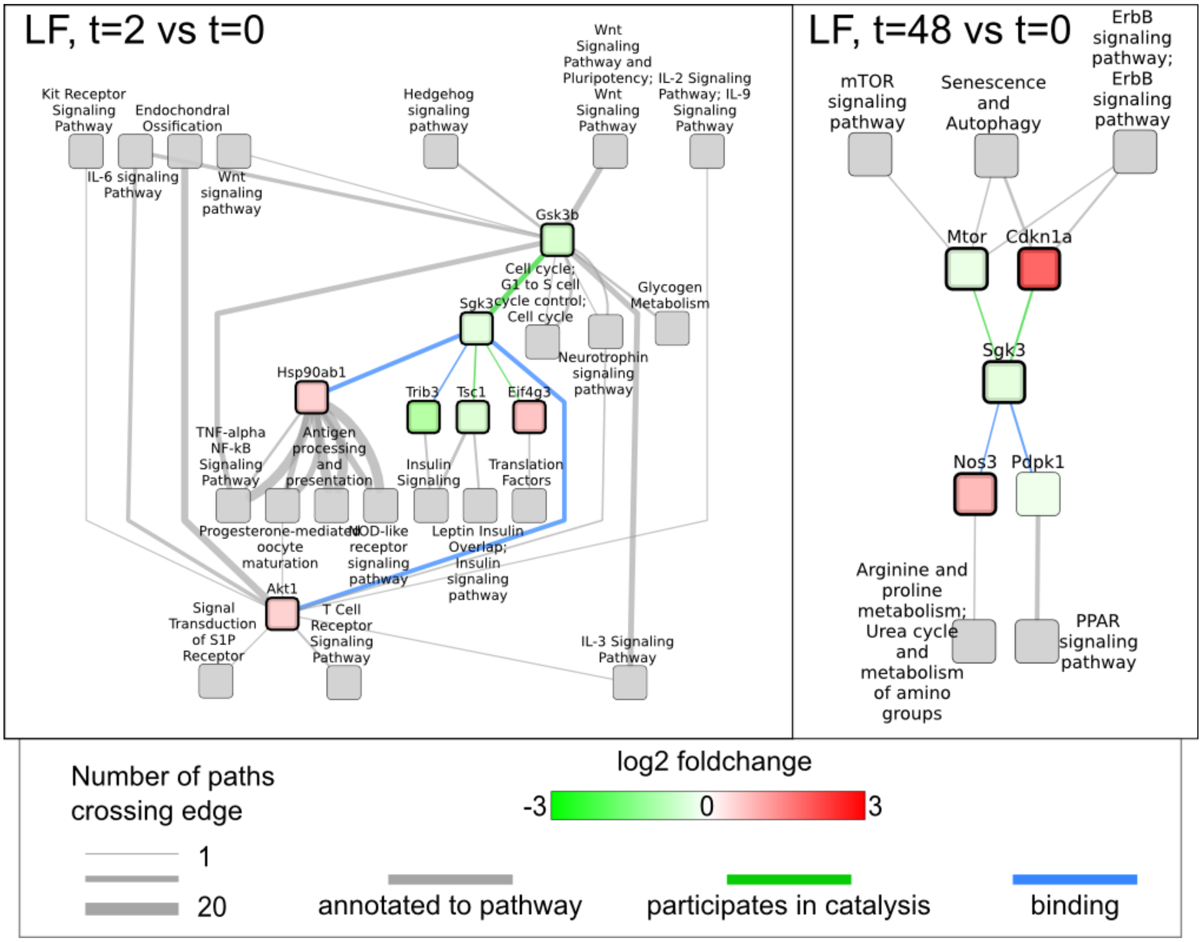

Supplementary figure 5: Interactions with Sgk3 that are part of identified indirect paths between pathways in the network for the response in LF at t=2 and t=48.

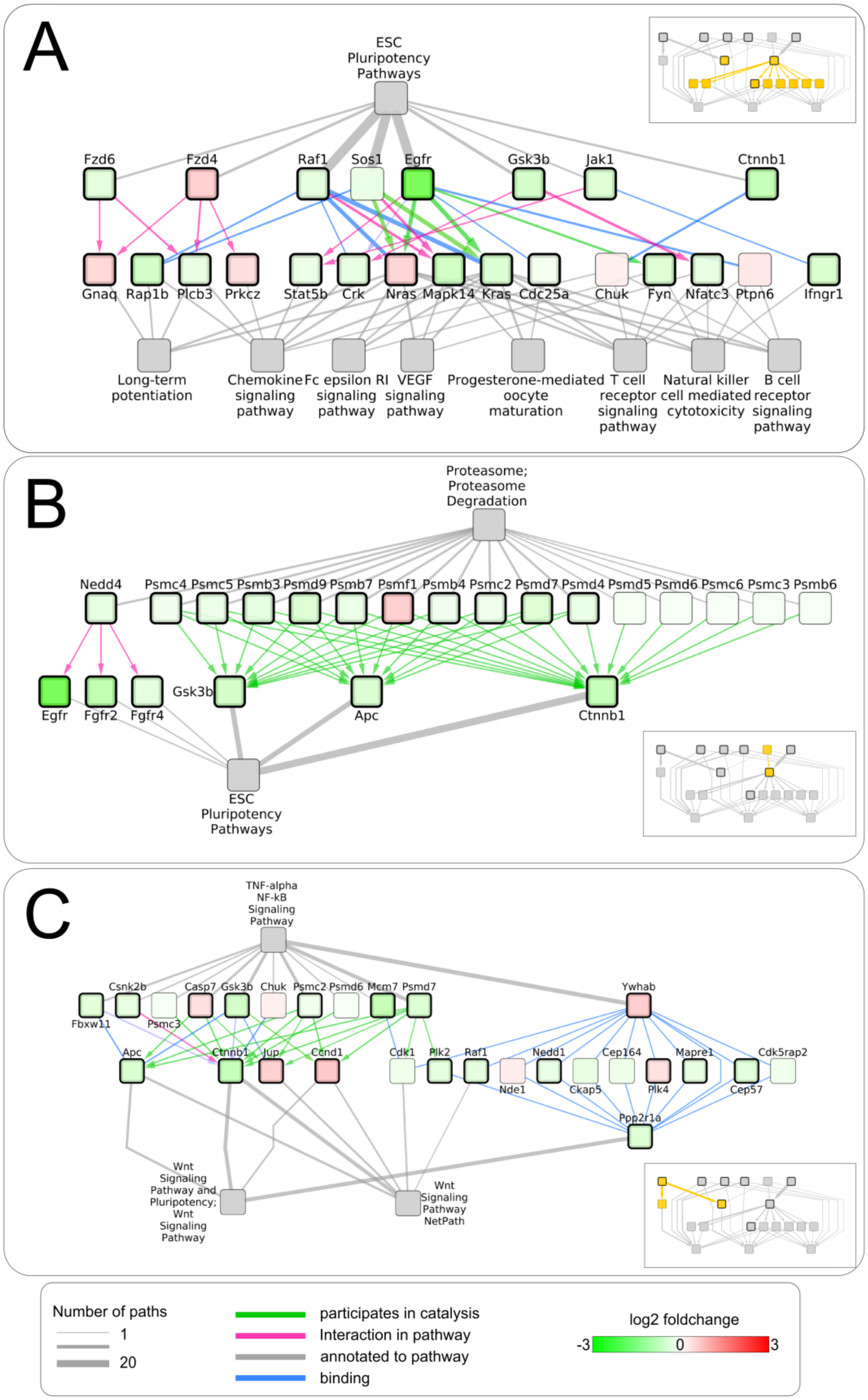

Supplementary figure 6: The protein interactions that compose several pathway interactions in the subgraph as shown in Figure 3A (see insets, included interactions are marked orange). Protein nodes have a thick border when they are significantly differentially expressed (q < 0.05). A: interactions between the ESC Pluripotency pathway and its neighbors, B: interaction between the Proteasome pathway and ESC Pluripotency pathway, C: interactions between the TNF-alpha NF-kB Signaling pathway and the two versions of the Wnt Signaling pathway.

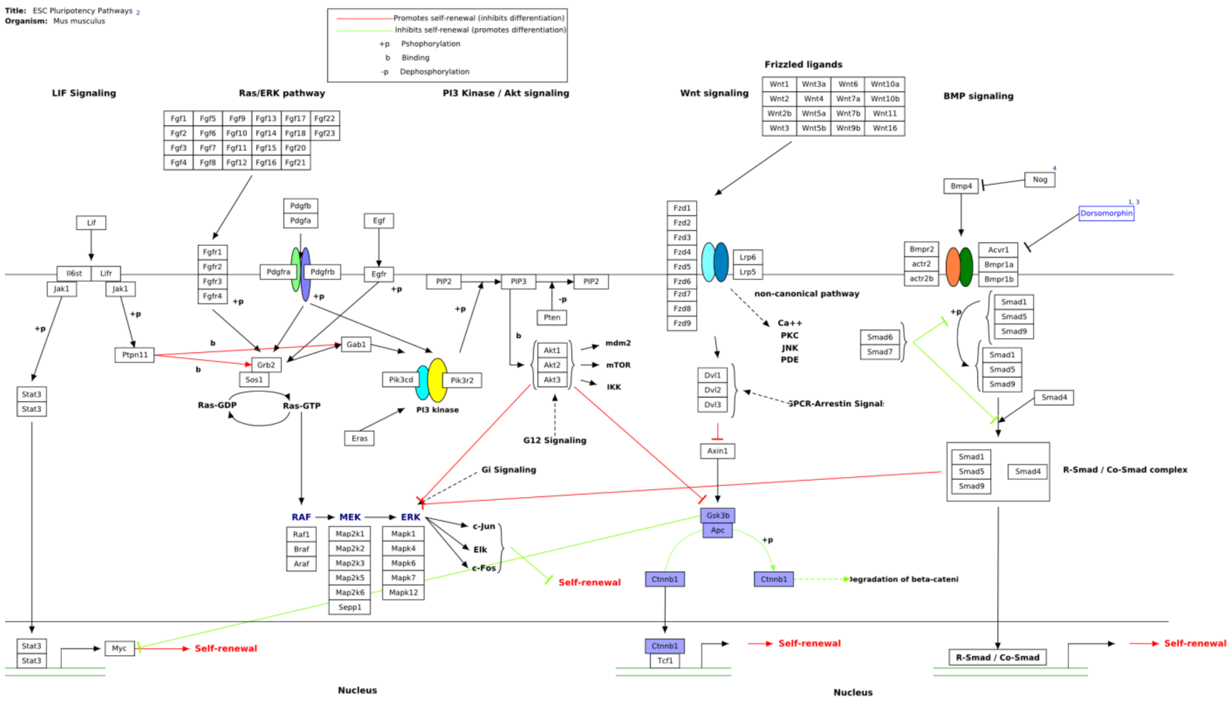

Supplementary figure 7: The diagram for the ESC Pluripotency pathways. Highlighted in blue are the proteins Apc, Ctnnb1 and Gsk3b which are identified in the interaction with the Proteasome pathway in the network for the comparison between diets at t=0.

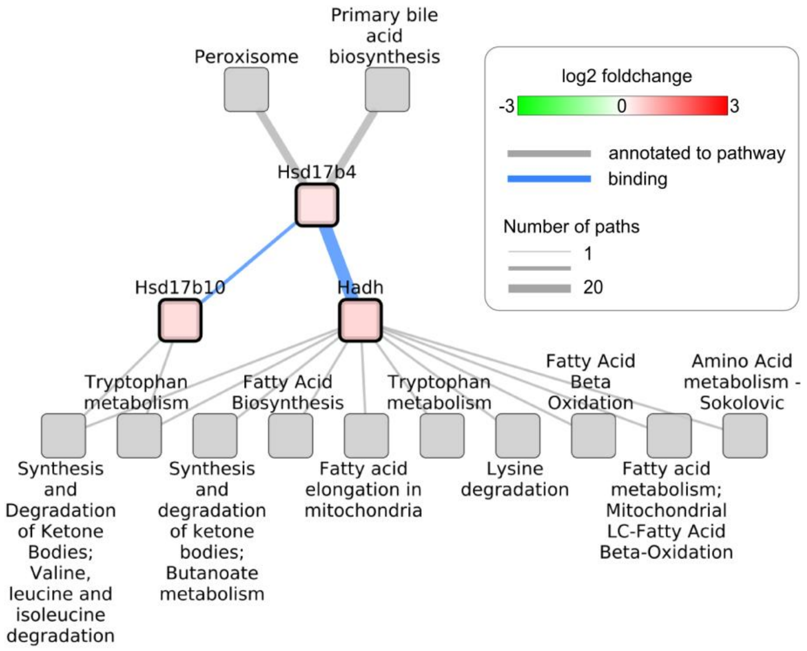

Supplementary figure 8: Protein interactions contributing to the pathways between the Primary Bile Acid Biosynthesis and Peroxisome pathways and several other metabolic pathways in the network for comparison between diets at t = 0. The three proteins are all significantly up-regulated in the HF group and their interactions are binding.

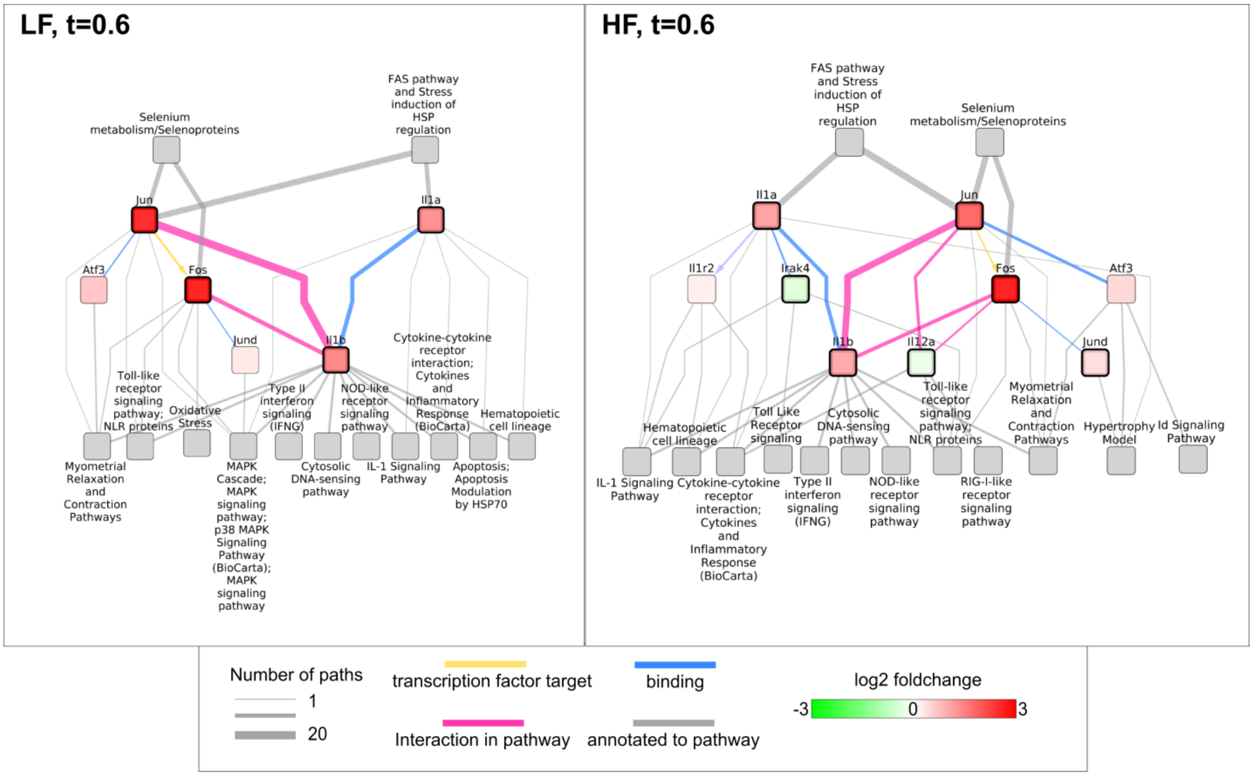

Supplementary Figure 9: Protein interactions playing a role in the interactions between pathways for the networks based on differential expression between t=0 and t=0.6 in the LF diet group (left) and the HF diet group (right).

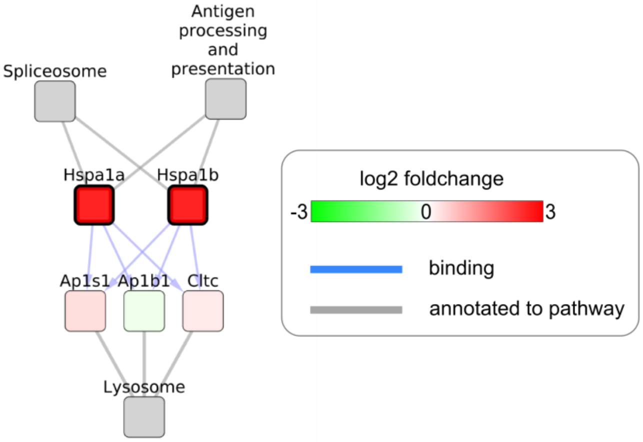

Supplementary figure 10: Protein interactions playing a role in the interactions between pathways in the component composed by the Antigen processing and presentation, the Spliceosome and Lysosome pathways for the network based on differential expression between t=0 and t=0.6 in the HF diet group.

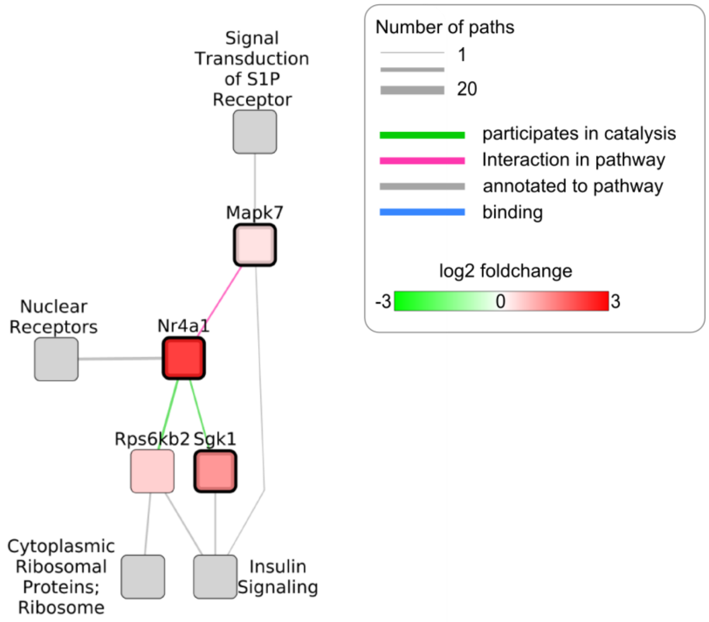

Supplementary figure 11: Protein interactions playing a role in the interactions between pathways in the component composed by the S1P Receptor, Nuclear Receptors, Cytoplasmic Ribosomal Proteins and Insulin Signaling pathways for the network based on differential expression between t=0 and t=0.6 in HF diet.

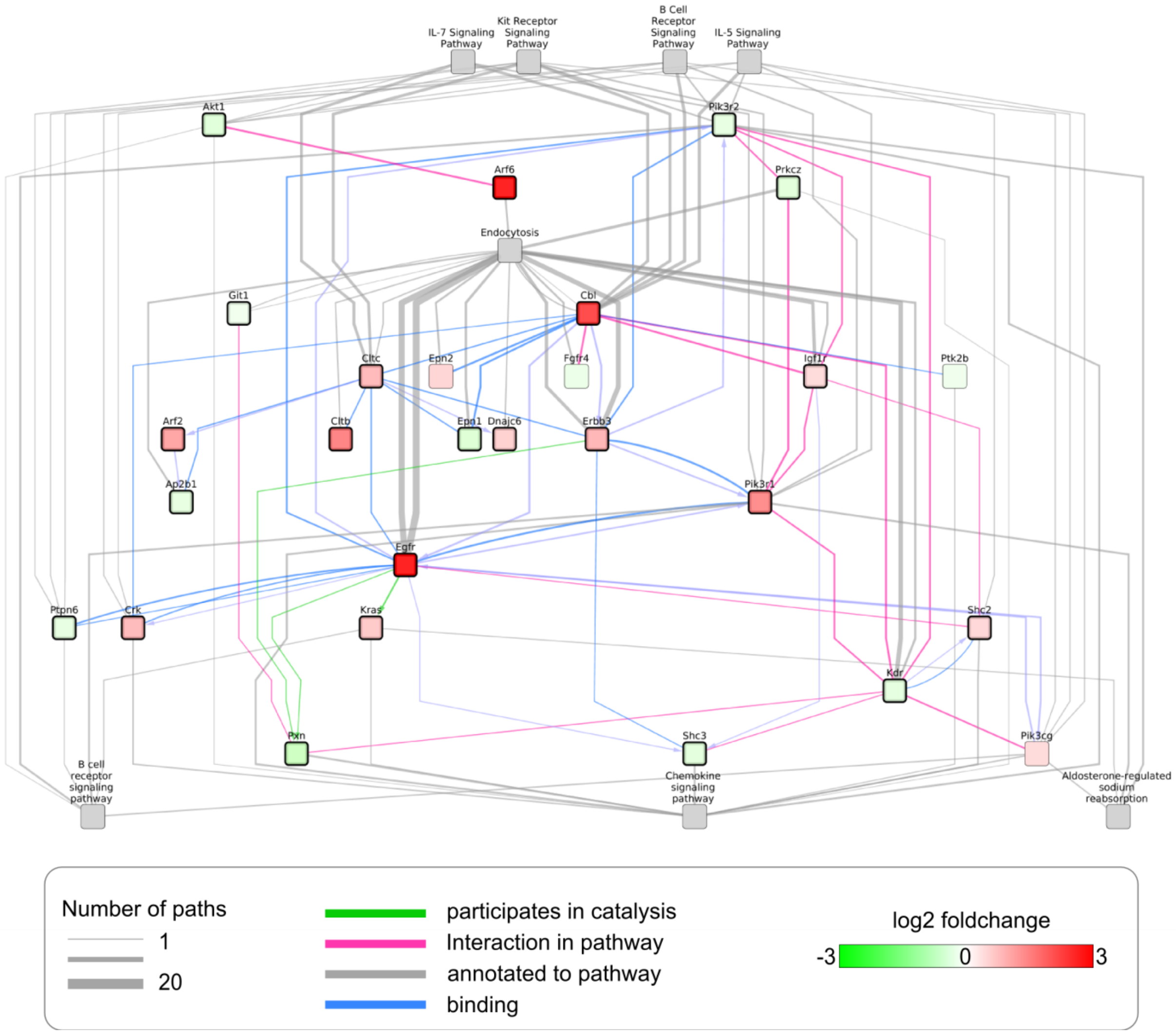

Supplementary figure 12: Protein interactions for the paths between the Endocytosis pathway and its neighbors in the network for differential expression between t=2 and t=0 in the HF diet group.

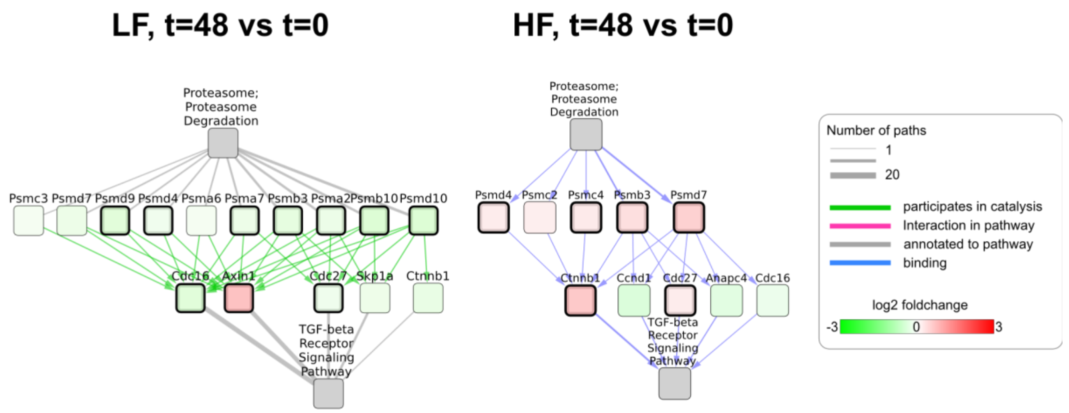

Supplementary figure 13: Proteins forming the paths between the Proteasome and TGF-beta Receptor Signaling pathways for the networks based on differential expression between t=48 and t=0 in the LF group (left) and HF group (right).

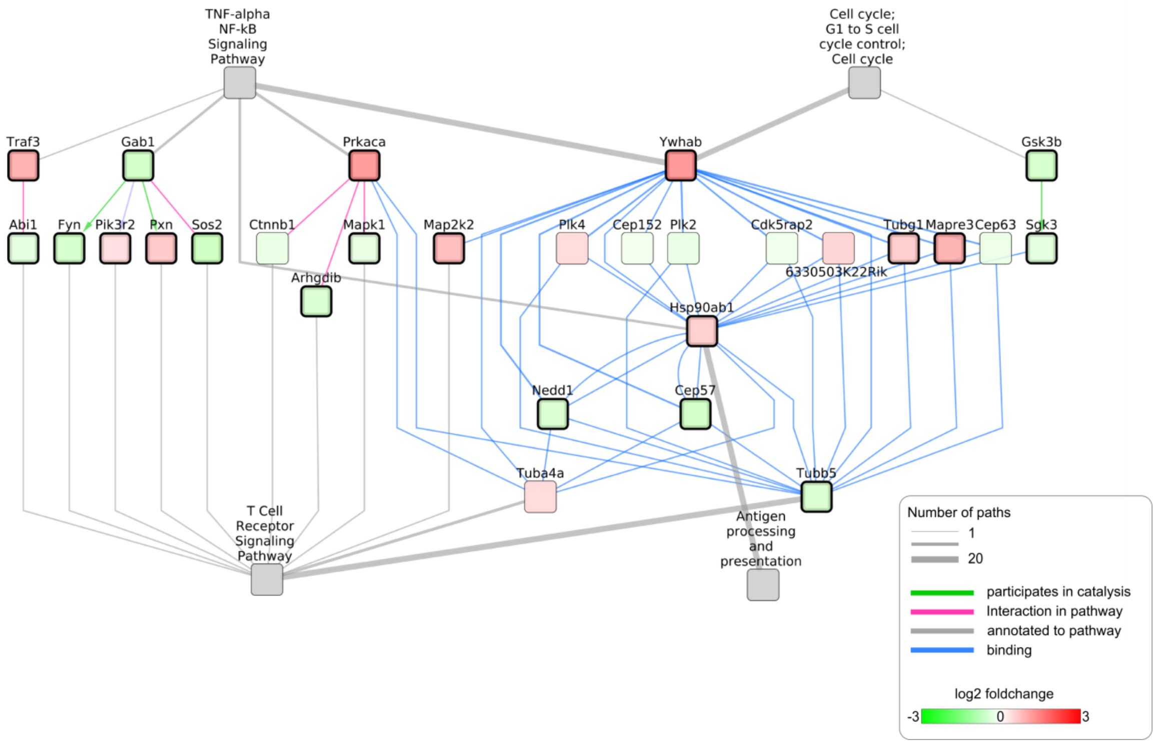

Supplementary figure 14: Paths between the Antigen processing and presentation, T Cell Receptor Signaling, Cell Cycle and TNF –alpha NF-kB pathways in the network for differential expression between t=48 and t=0 in the LF diet.

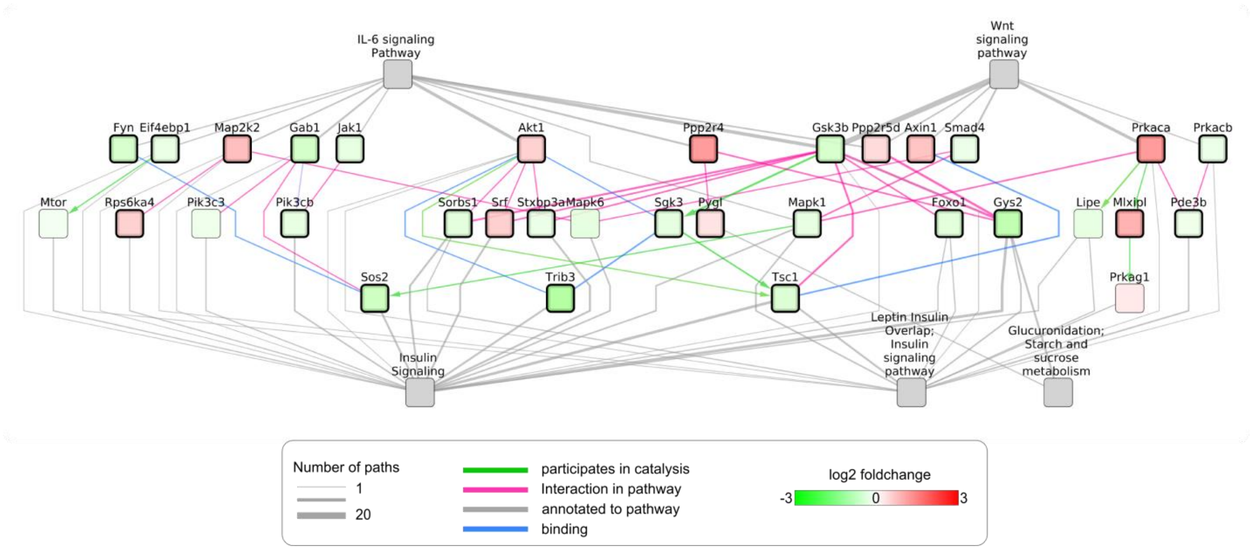

Supplementary figure 15: Paths between the pathways in the second order neighborhood of the Insulin Signaling pathway in the network for differential expression between t=48 and t=0 in the LF diet.

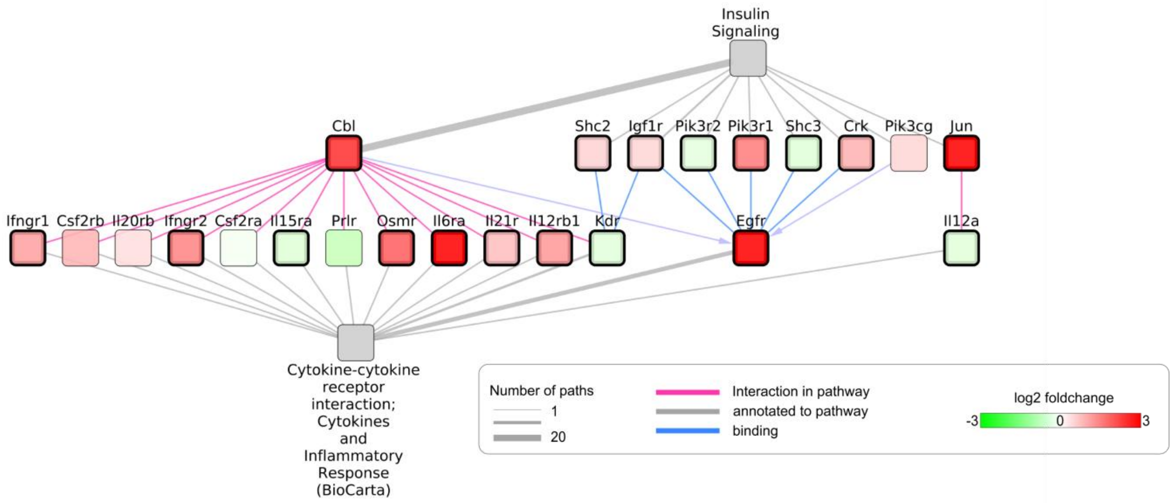

Supplementary figure 16: Interaction between the Insulin and Cytokine-cytokine receptor interaction pathways for the network based on differential expression between t=0 and t=2 in the HF group.

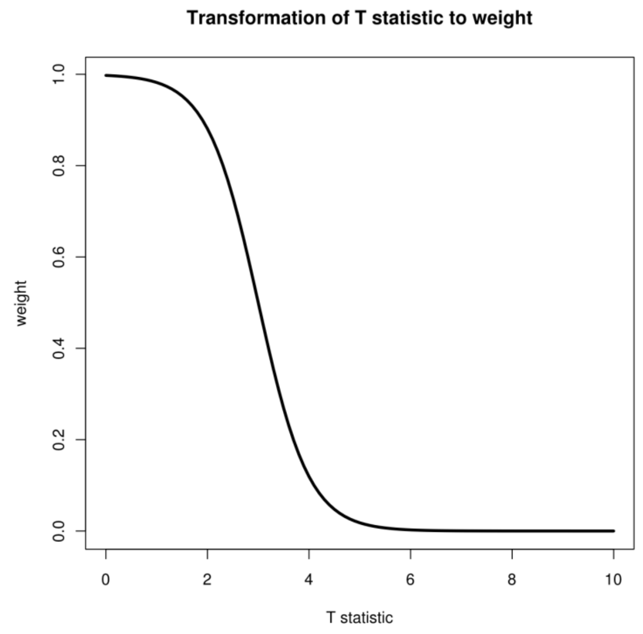

Supplementary figure 17: Transformation of T statistic to edge weights using the sigmoid function, with parameters μ=3 and α=2.

Supplementary tables

	Nr. paths	Involved in interactions with pathways
**HF vs LF, t = 0**		
Kras	109	Dorso-ventral axis formation (15), FAS pathway and Stress induction of HSP regulation (14), ESC Pluripotency Pathways (13), 47 more.
Raf1	96	ESC Pluripotency Pathways (13), FAS pathway and Stress induction of HSP regulation (11), Oxidative Damage; Apoptosis (11), 45 more.
Nras	85	FAS pathway and Stress induction of HSP regulation (11), Oxidative Damage; Apoptosis (11), Long-term depression (10), 34 more.
Ctnnb1	64	Ptf1a related regulatory pathway (12), Dorso-ventral axis formation (10), Proteasome; Proteasome Degradation (7), 43 more.
Sos1	62	ESC Pluripotency Pathways (13), Dorso-ventral axis formation (10), FAS pathway and Stress induction of HSP regulation (7), 40 more.
**LF, t = 0.6 vs t = 0**		
Jun	25	FAS pathway and Stress induction of HSP regulation (11), Selenium metabolism/Selenoproteins (11), Oxidative Stress (3), 14 more.
Il1b	20	FAS pathway and Stress induction of HSP regulation (10), Selenium metabolism/Selenoproteins (9), Toll-like receptor signaling pathway; NLR proteins (3), 10 more.
Fos	17	Selenium metabolism/Selenoproteins (10), FAS pathway and Stress induction of HSP regulation (4), Oxidative Stress (3), 13 more.
Il1a	11	FAS pathway and Stress induction of HSP regulation (10), Toll-like receptor signaling pathway; NLR proteins (2), 10 more.
Atf3	5	Selenium metabolism/Selenoproteins (3), Hypertrophy Model (2), Myometrial Relaxation and Contraction Pathways (2), 3 more.
**LF, t = 2 vs t = 0**		
Mapk1	21	Signal Transduction of S1P Receptor (6), Cytoplasmic Ribosomal Proteins; Ribosome (5), Vasopressin-regulated water reabsorption (5), 18 more.
Prkacb	16	Taste transduction (5), Vasopressin-regulated water reabsorption (5), Hypothetical Network for Drug Addiction (3), 13 more.
Rps6ka1	10	Signal Transduction of S1P Receptor (6), Cytoplasmic Ribosomal Proteins; Ribosome (5), 9 more.
Hspa1b	8	Spliceosome (5), SNARE interactions in vesicular transport (3), Antigen processing and presentation (2), 5 more.
Mapk6	6	Signal Transduction of S1P Receptor (6), 6 more.
**LF, t = 48 vs t = 0**		
Prkaca	48	Endochondral Ossification (9), Antigen processing and presentation (9), T Cell Receptor Signaling Pathway (8), 25 more.
Cep57	44	Antigen processing and presentation (11), T Cell Receptor Signaling Pathway (9), Endochondral Ossification (8), 16 more.
Nedd1	44	Antigen processing and presentation (11), T Cell Receptor Signaling Pathway (9), Endochondral Ossification (8), 16 more.
Mapre3	44	Antigen processing and presentation (11), T Cell Receptor Signaling Pathway (9), Endochondral Ossification (8), 16 more.
Tubg1	44	Antigen processing and presentation (11), T Cell Receptor Signaling Pathway (9), Endochondral Ossification (8), 16 more.
**HF, t = 0.6 vs t = 0**		
Jun	34	Hypertrophy Model (13), Selenium metabolism/Selenoproteins (10), FAS pathway and Stress induction of HSP regulation (8), 19 more.
Fos	28	Hypertrophy Model (15), Selenium metabolism/Selenoproteins (9), T cell receptor signaling pathway (4), 18 more.
Il1b	23	FAS pathway and Stress induction of HSP regulation (8), Selenium metabolism/Selenoproteins (6), Hypertrophy Model (4), 11 more.
Il1a	17	FAS pathway and Stress induction of HSP regulation (9), Hypertrophy Model (4), Toll Like Receptor signaling (4), 9 more.
Atf3	15	Hypertrophy Model (13), Selenium metabolism/Selenoproteins (3), Myometrial Relaxation and Contraction Pathways (2), 11 more.
**HF, t = 2 vs t = 0**		

Pik3r1	60	Cytokine-cytokine receptor interaction; Cytokines and Inflammatory Response (BioCarta) (14), Dorso-ventral axis formation (12), Aldosterone-regulated sodium reabsorption (7), 35 more.
Pik3r2	51	Dorso-ventral axis formation (13), Cytokine-cytokine receptor interaction; Cytokines and Inflammatory Response (BioCarta) (12), Aldosterone-regulated sodium reabsorption (7), 32 more.
Egfr	45	Cytokine-cytokine receptor interaction; Cytokines and Inflammatory Response (BioCarta) (15), Dorso-ventral axis formation (15), Endocytosis (7), 33 more.
Akt1	41	Endochondral Ossification (6), EPO Receptor Signaling (6), Signal Transduction of S1P Receptor (6), 31 more.
Pik3cg	33	Dorso-ventral axis formation (12), Cytokine-cytokine receptor interaction; Cytokines and Inflammatory Response (BioCarta) (11), Endocytosis (6), 22 more.
**HF, t = 48 vs t = 0**		
Pik3r1	39	Axon guidance (8), Tight junction (8), IL-4 signaling Pathway (5), 33 more.
Kras	37	Axon guidance (8), Tight junction (8), IL-4 signaling Pathway (5), 33 more.
Ctnnb1	35	Proteasome; Proteasome Degradation (10), Tight junction (8), Ptf1a related regulatory pathway (6), 25 more.
Prkca	22	Keap1-Nrf2 (5), Axon guidance (4), Phosphatidylinositol signaling system; Inositol phosphate metabolism (4), 20 more.
Mapk14	18	Axon guidance (6), IL-4 signaling Pathway (5), Dorso-ventral axis formation (4), 15 more.

Supplementary Table 1: Top 5 proteins involved in most significant pathway interactions for each comparison. The last column displays the pathways in which interactions the protein is most involved, with the number of interactions in parentheses.
