# Supplementary material for: Exploring pathway interactions in insulin resistant mouse liver
Source: BMC Syst Biol. 2011 Aug 15;5:127. doi: 10.1186/1752-0509-5-127 (PMC3169508; doi:10.1186/1752-0509-5-127)
Supplement: Additional file 3 — Scripts and input data. Contains instructions on how to obtain the scripts, utilities and formatted input data that was used in this analysis. [file 1752-0509-5-127-S3.PDF]

Scripts and input data

Source code of all scripts, as well as input data and Cytoscape and R session files of the analysis results are available from:

http://code.google.com/p/tkelder/wiki/PathwayInteractions
